# Network indices show heat wave‐induced fragility in experimental food webs: Operational insights for ecosystem management

**DOI:** 10.1002/eap.70277

**Published:** 2026-09-08

**Authors:** Maysa Ito, Anna Waffender, Marco Scotti

**Affiliations:** ^1^ GEOMAR Helmholtz Centre for Ocean Research Kiel Kiel Germany; ^2^ National Research Council of Italy, Institute of Biosciences and Bioresources Sesto Fiorentino Italy

**Keywords:** detritivory, ecosystem‐based management, EU Marine Strategy Framework Directive, information theory indices, mesocosms, western Baltic Sea

## Abstract

Emerging global change imposes challenges for marine ecosystems placing them at the verge of collapse. The identification of early signs of ecosystem collapse is crucial to assist adaptive management. Shifts in the ecosystems are preceded by rewiring of ecological networks. Environmental assessment still lacks indicators that quantify the state of ecological processes (e.g., trophic interactions). Thus, evaluating ecosystems using network indices is appropriate, although their responses to stress factors are not fully resolved. Network indices have been proposed to identify the good environmental status, but experimental evidence of their behavior under disturbance remains limited. We used data from a mesocosm experiment simulating different frequencies of sublethal heat waves (three sequential events across summer—3HW, and a single abrupt event at the end of summer—1HW) to construct carbon flow networks and calculate information theory indices, testing their responsiveness to disturbance. A comparison with ecological attributes was conducted to interpret the trends of network indices and assess whether these indices provide complementary insights to the ecological attributes. Our results show that, even without ecosystem collapse, carbon flow networks representing communities exposed to heat waves exhibited reduced link‐weighted connectance and lower internal carbon‐use efficiency compared to the control. These changes were associated with increased detritivory under both heat wave treatments, along with higher respiration losses and shorter average residence time of carbon after 1HW. Information theory indices proved responsive to disturbance, detecting early signs of stress that were not captured by changes in ecological attributes alone. Our findings show that information theory indices respond consistently to heat waves. Grounded in robust theoretical foundations, our study advances the operationalization of these indices for ecosystem‐based management.

## INTRODUCTION

Climate change is one of the greatest threats to marine biodiversity (Pörtner et al., 2023). Besides causing an increase in the average temperature, it also leads to a higher occurrence of extreme events, such as marine heat waves (MHWs), which have been intensifying and becoming more frequent (Frölicher & Laufkötter, 2018). In some ocean regions, MHWs have exceeded the lethal temperature threshold of vulnerable organisms, resulting in massive mortality (Piatt et al., 2020). The mortality of habitat‐forming species was followed by a decline in biodiversity, having implications for economic activities (Rogers‐Bennett & Catton, 2019; Smale et al., 2019). Evaluating the consequences of MHWs becomes particularly challenging when these extreme events are sublethal and remain within species' tolerance levels. Sublethal MHWs modulate the physiology of the organisms and alter their feeding rates, with cascading effects that can cause species' biomass to either increase or decrease, making it difficult to determine the net ecosystem response (Ito et al., 2024). One solution is to employ network indices, which can assess whether sublethal MHWs influence ecosystem structure, shift the balance among carbon circulation pathways, and alter ecosystem functioning (Ulanowicz, 1986, 2004).

Network indices have been applied to track the temporal development of natural ecosystems (Scotti et al., 2022), inform about the overall impact of increasing human pressure (e.g., fish extraction; Ito et al., 2023), and evaluate the cumulative effect of anthropogenic stressors (Brito et al., 2024). They have been proposed as a suitable tool for supporting the sustainable management of marine resources (Tam et al., 2017) and operationalizing the EU Marine Strategy Framework Directive (MSFD, 2008, 2017) through the quantification of the abstract concept of Good Environmental Status (GES; Safi et al., 2019). The efficacy of these indices lies in their responsiveness to disturbance of distinct types and intensity. Although in silico applications exploring the relationships between various levels of stress and indices' responses exist, they often consider fisheries as the main factor of disturbance (Ito et al., 2023; Samhouri et al., 2009). Some experimental studies investigated instead the impact of warming and pesticides on food webs, estimating interaction strengths between trophic groups and a few network properties (Polazzo et al., 2022, 2023). Nevertheless, network indices have not been comprehensively explored using experimental approaches.

Multiple mechanisms explain how climate change reshapes the structure of marine food webs and modifies ecosystem functioning. Ocean warming and acidification alter community assembly, reduce energy transfer efficiency between producers and consumers, and thereby simplify food webs (Ullah et al., 2018). Climate change shifts species' spatial distribution, composition, and relative abundance, thus impacting food web connectance and the strength of trophic chains across habitats (Kortsch et al., 2015). Feeding intensity varies nonlinearly across temperature gradients, and species reach optimal growth and metabolic rates at different temperatures, complicating forecasts of how warming regulates the balance between bottom‐up and top‐down forces in the food web (Ito et al., 2019). Repeated and extreme MHWs shorten biomass residence time in the ecosystem and restructure energy fluxes by increasing food web connectance (Gomes et al., 2024). A substantial knowledge gap remains regarding how sublethal MHWs at different frequencies influence carbon distribution among food web interactions. There is a need to develop approaches capable of detecting even slight changes in carbon flow circulation following exposure to heat waves. This objective can be achieved by applying network analysis, which has been proposed as a suitable method for GES assessment as it integrates biotic and abiotic ecosystem components (McQuatters‐Gollop et al., 2022). Nevertheless, the indices require further refinement to improve interpretation and communication with stakeholders.

This study evaluates whether network indices characterizing the structure of a marine benthic food web disturbed by sublethal heat waves may reveal signs of stress. Ito et al. (2024) demonstrated that heat waves at varying frequencies modify carbon fluxes, but how they reconfigure the architecture of carbon pathways remains unknown. Data were obtained from a mesocosm experiment in which a temperate benthic ecosystem was exposed to three temperature regimes: no heat waves (0HW), a single summer heat wave (1HW), and three sequential heat waves across spring and summer (3HW). Our hypothesis is that exposure to heat waves simplifies food web structure (Polazzo et al., 2023) by increasing carbon dissipation (Göbeler et al., 2025) and strengthening consumers' interactions with detritus. This study has implications for determining the GES using indices derived from information theory, which may be applied in environmental assessment and support management planning.

## MATERIALS AND METHODS

### Mesocosms

The experiment was conducted at the Kiel Outdoor Benthocosms (KOB; Wahl et al., 2015) from 6 May to 20 August 2015. The facility is located on the Kiel Fjord (54.33 N, 10.15 E) and comprises 12 tanks, each with a capacity of 1500 L and a base area of 1.53 m^2^. These units receive a continuous inflow of unfiltered seawater (1800 L day^−1^) directly from the Kiel Fjord, allowing filamentous algae, sediment infauna, and epifauna larvae to enter and settle in the tanks. Heat waves were simulated by elevating the water temperature during the experiment.

### Heat waves

**FIGURE 1:**
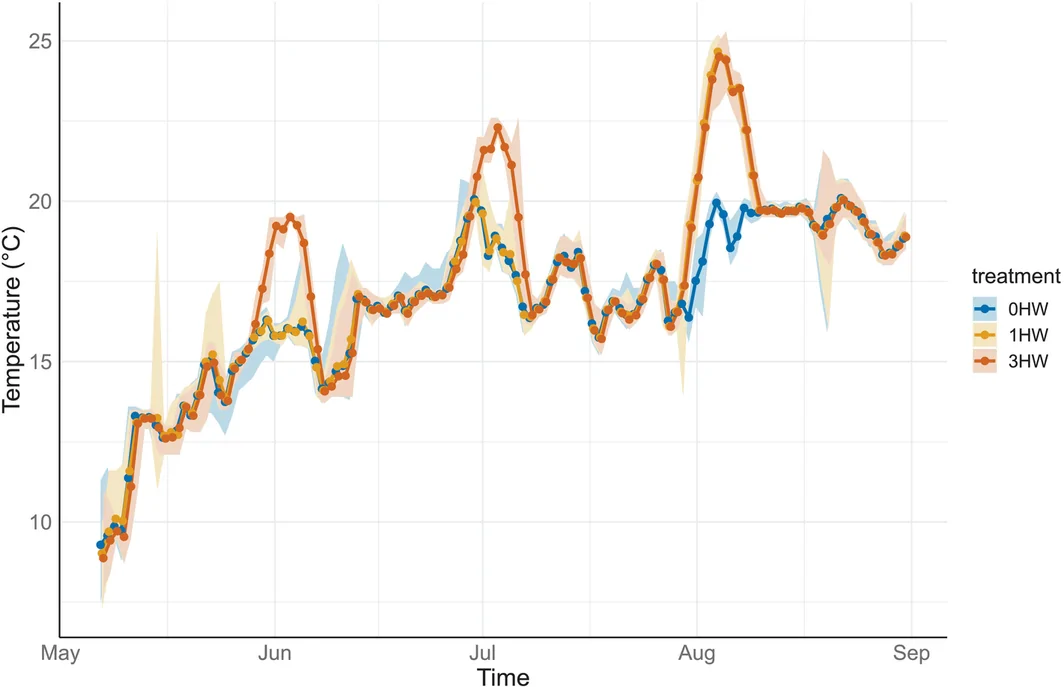
Temperature profiles applied to 12 mesocosm tanks: no heat waves (0HW), one late‐summer heat wave (1HW), or three sequential heat waves spanning late spring–summer (3HW); four replicates per treatment. The experiment lasted 106 days, starting on 6 May and ending on 20 August 2015. Colored circles refer to daily mean temperatures. Shaded areas indicate the daily minimum and maximum temperatures recorded for each treatment.

To determine the temperature profile of the control treatment, as well as the amplitude and duration of heat waves, a generalized additive mixed model (GAMM) was applied to 15 years of temperature data (2000–2014) collected at 1.5 m depth in the Kiel Fjord (54.33 N, 10.15 E). The model was used to extract both long‐term trends and within‐year (seasonal) variation. The analysis identified 2009 as the year with the lowest number of average daily temperatures outside the 95% CI of the GAMM seasonal term. Therefore, we used the 2009 temperature profile as the no heat wave treatment (0HW) and baseline to apply heat waves. Warming treatments consisted of one heat wave at the end of summer (1HW) and three heat waves along end of spring and through summer (3HW). All treatments were replicated four times. Each heat wave lasted 9 days and peaked at sublethal temperatures, remaining below thresholds that could have caused mass mortality in the experimental tanks. Heat waves in June and July simulated for the 3HW treatment attained temperature values of 3.6°C higher than in the 0HW and 1HW treatments. The warming phase lasted 3 days and the increase rate was 1.2°C day^−1^, which represents the highest average daily increase detected for heat waves from 1 May to 30 September in the Kiel Fjord time series. The temperature peak lasted 4 days, which is the median of days between heat waves longer than 2 days and cooling phases. The cooling phase lasted 2 days with temperature decrease ranging from −0.8 and −2.0°C day^−1^, which are the minimum and maximum average daily slopes in the Kiel Fjord time series, respectively. The last heat wave was the most intense and was applied to both the 1HW and 3HW treatments in August. It corresponded to the average of the four most extreme heat waves in the years 2000–2014, with the peak reaching 5.2°C above the 0HW temperature. The warming average rate was 1.7°C day^−1^ for 3 days, the peak was maintained for 4 days, and the cooling phase lasted 2 days (Figure 1). Further details on the model used to design the heat waves are in the supporting information of both Ito et al. (2024) and Pansch et al. (2018).

### Community composition

The organisms introduced into the tanks were collected between 4 and 6 May 2015 in the western Baltic Sea. The seagrass *Zostera marina* was collected from Falkenstein (54.39 N, 10.19 E) at 2–3 m depth. Plants were transported within 2 h to the KOB. Sediment was collected from the same seagrass meadow, sieved through a 2000‐μm mesh and homogenized, then used to fill 5‐L plastic boxes or 1‐L plastic beakers into which the seagrasses were planted. Each tank received 11 boxes of six plants and 10 beakers of two plants. The macroalga *Fucus vesiculosus* and the associated mesograzers (*Gammarus* spp., *Idotea balthica*, and *Littorina littorea*) were collected from 0.5 m depth in Bülk (54.45 N, 10.20 E). Five specimens of *F. vesiculosus*, that is, two small (90.27 ± 24.32 g wet mass, WM), two medium (202.81 ± 44.92 g WM), and one large (415.62 ± 77.03 g WM), were placed in each tank. Equal numbers of amphipods *Gammarus* spp. (50 individuals), the isopod *I. balthica* (20 individuals) and the gastropod *L. littorea* (80 individuals) were added to each tank. Although the initial community was composed of primary producers and mesograzers only, organisms settled in the tanks during the experiment and the analysis includes other species such as bivalves and polychaetes.

### Network construction

#### Compartments

The living compartments (trophic groups) considered in this study include three primary producers, that is, seagrasses (*Z. marina*), bladderwracks (*F. vesiculosus*), and filamentous algae; three consumers added at the beginning of the experiment, that is, isopods omnivores (*I. balthica*), amphipods omnivores (*Gammarus* spp.), and gastropods omnivores (*L. littorea*); and other consumers that settled during the experiment, that is, isopods detritivores, amphipods detritivores, gastropods detritivores, bivalves filter‐feeders, polychaetes omnivores, and polychaetes detritivores (Appendix S1: Table S1). In addition, a nonliving compartment representing the particulate organic carbon (POC) was considered. At the end of the experiment, organisms were collected from the tanks for identification and biomass quantification (Appendix S1: Section S1).

#### Carbon fluxes

**FIGURE 2:**
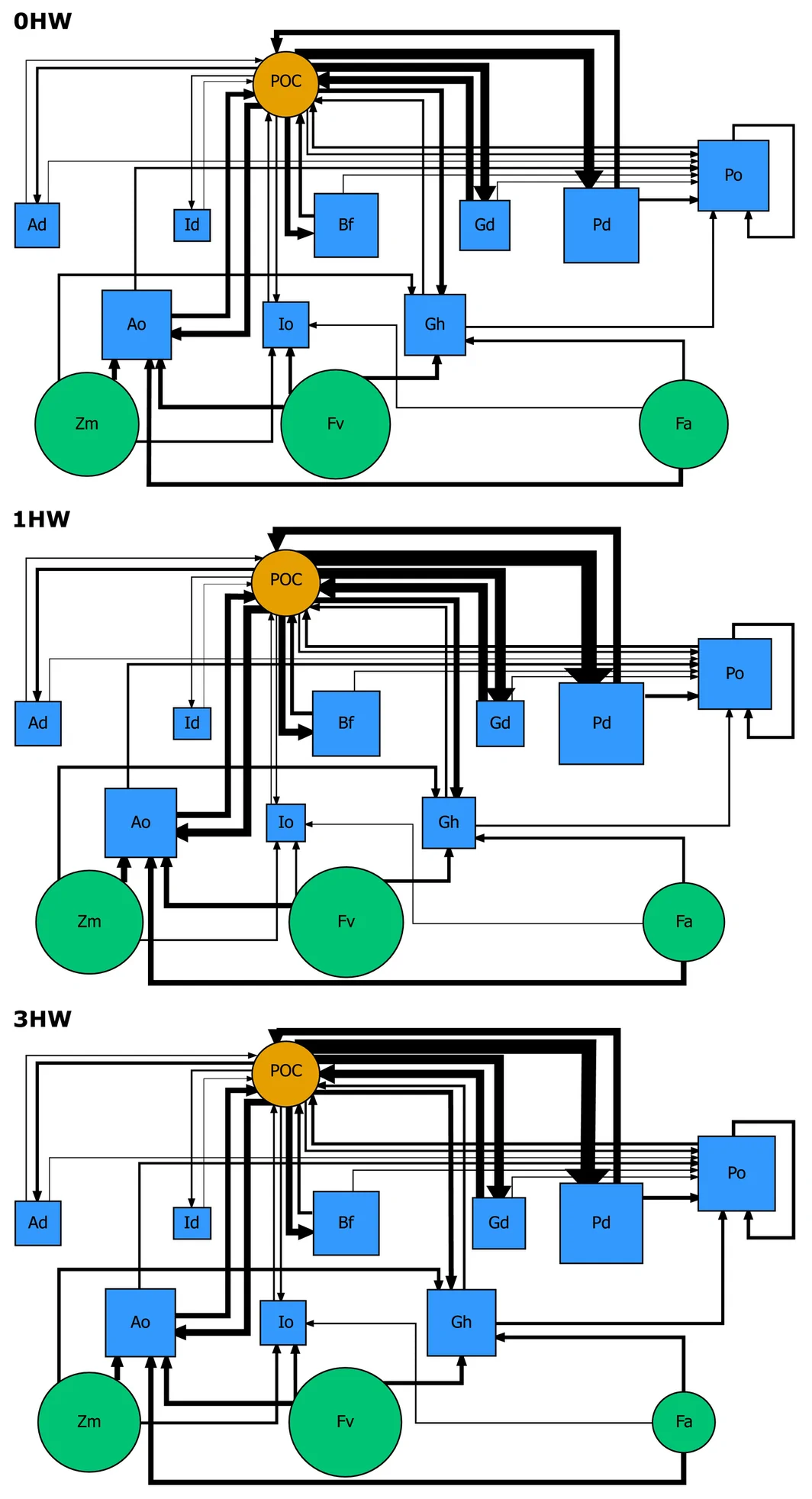
Carbon flow networks in the experimental ecosystems under no heat waves (0HW), one late‐summer heat wave (1HW), and three sequential heat waves spanning late spring–summer (3HW) treatments. Node size is proportional to the log‐transformed average biomass of the compartment in each treatment while colors distinguish between primary producers (green), consumers (blue), and nonliving node (yellow). Arrows indicate the direction of carbon circulation, from resources to consumers, with their square root‐transformed thickness representing the magnitude of carbon transfer. Ad, amphipods detritivores; Ao, amphipods omnivores; Bf, bivalves filter‐feeders; Gd, gastropods detritivores; Gh, gastropods omnivores; Fa, filamentous algae; Fv, *Fucus vesiculosus*; Id, isopods detritivores; Io, isopods omnivores; Pd, polychaetes detritivores; Po, polychaetes omnivores; POC, particulate organic carbon; Zm, *Zostera marina*.

Networks of carbon fluxes, expressed in milligrams of carbon per square meter per day (Figure 2), were constructed by combining empirical data from the mesocosms with information obtained from the literature. Four types of fluxes account for external input (gross primary production, GPP), dissipation (respiration), export outside ecosystem's boundaries (e.g., floating talli of bladderwrack) and exchanges among compartments (e.g., trophic interactions). We quantified the magnitude of carbon fluxes from compartment biomass: for primary producers using photosynthesis, respiration, and export rates; for consumers using production and respiration rates, assimilation efficiency, and feeding preferences.

##### Gross primary production, respiration, and export

GPP is the carbon input entering the food web through the primary producers. It was calculated by summing the macrophytes' net primary production (NPP) rate, measured during light incubation, and their respiration rate, measured with dark incubation (Appendix S1: Section S2). Respiration constitutes a loss from the network and represents unusable carbon leaving the ecosystem (Appendix S1: Section S2). By contrast, exports denote usable carbon transferred beyond the boundaries of the system under study. After the last heat wave, we quantified the respiration rates of omnivorous isopods, amphipods, and gastropods (Appendix S1: Section S3).

##### Intercompartmental exchanges

Consumption (*Q*) by a heterotrophic compartment represents the total carbon flow into that trophic group. Consumers' production to biomass (*P*/*B*; Appendix S1: Table S2) and respiration to biomass (*R*/*B*; Appendix S1: Table S3) ratios served to calculate the gross production (GP) starting from the biomass of the compartment (*B*). GP was then divided by the assimilation efficiency (α; Appendix S1: Table S2) to determine *Q* (Equation 1).

(1) $$\frac{\left((P/B)\times B)+((R/B)\times B\right)}{\alpha }=\frac{\mathrm{GP}}{\alpha }=Q$$

All parameters were obtained from the literature, except the respiration rates of omnivorous isopods, amphipods, and gastropods, which were measured in incubation chambers during the experiment. When more species were included in a trophic group, the compartment‐level metabolic parameters (*P*/*B*, *R*/*B*, and α) were calculated as the biomass‐weighted average of the species' parameters, using each species' proportion of the compartment's total biomass. Literature‐derived rates were rescaled to 20°C (control) and 25°C (final heat wave) using Q10 values (Baird et al., 2004; Appendix S1: Table S4), which quantify the proportional change in metabolic rate associated with a 10°C temperature increase. Assimilation efficiencies found in the literature also accounted for the different temperatures of the treatments. Egestion (*E*) was estimated as the difference between total consumption and gross production (GP) (Equation 2); it corresponds to the strength of carbon flows from consumers to POC.

(2) Q−GP=E

Intercompartmental flows indicate carbon exchanges between the system's compartments. The presence of trophic interactions between compartments was determined based on a literature of reported prey items (Appendix S1: Table S5). When multiple resources were ingested, the magnitude of each flow was calculated by apportioning each trophic group's total consumption according to feeding preferences determined by stable isotope analysis (SIA) (Appendix S1: Section S4).

### Network analysis and ecosystem attributes

#### Information theory indices

To characterize ecosystem‐level responses to heat waves, we calculated a suite of information theory indices from the ecological network analysis (ENA) toolkit (Ulanowicz, 1986; Appendix S1: Table S6). These metrics are ideal because they do not require steady‐state conditions to be applied (Ulanowicz, 2004) and their responses have been linked to disturbance dynamics (Rutledge et al., 1976; Scotti et al., 2022; Appendix S1: Section S5). Ecosystem size was quantified by summing all carbon flows (total system throughput, TST). Flow diversity (*H*) was decomposed into two constitutive terms expressing the level of flow organization (average mutual information, AMI) and the degrees of freedom (df) pertaining to carbon circulation (residual diversity, Hc) (Equation 3). AMI is often referred to as a proxy for ecosystem development. Hc informs instead about the network's redundant routes, which increase with the number of connections and the homogeneity in the intensity of carbon flows; Hc decreases when carbon flows in the ecosystem are regulated by a few compartments and transferred via a few links.

(3) H=AMI+Hc

Once these terms are multiplied by the TST, the upper limit to ecosystem development (development capacity, DC), an index expressing growth and development (ascendency, *A*), and a measure on the carbon exchanged through redundant pathways (overhead, *O*) are obtained (Equation 4).

(4) $$H\times \mathrm{TST}=\left(\mathrm{AMI}+\mathrm{Hc}\right)\times \mathrm{TST}=\mathrm{DC}=A+O$$

The overhead is the counterpart of the ascendency and quantifies the system's capacity to withstand disturbance. It is derived from the sum of components that report the redundancy retained across four types of flows: overhead on imports (OI), which informs on the evenness of the carbon that enters the system (e.g., photosynthesis); overhead on exports (OE), for the df exhibited by usable carbon leaving the system (e.g., floating bladderwrack talli); dissipative overhead (OD), which characterizes whether the system's compartments contribute in a uniform way to respiration losses; and redundancy (*R*), which relates to the df shown by the internal connections transferring carbon among compartments (Equation 5). The terms composing the overhead (*O*) quantify different aspects of system resilience. OI reflects the resilience of carbon sources and increases with both the magnitude of external inputs and the evenness of flows among primary producers. Redundancy (*R*) captures the diversity of internal fluxes, which positively correlates with pathway multiplicity and enables the ecosystem to respond to disturbance. In contrast, increases in respiration and export fluxes beyond the system's boundaries, especially when accompanied by a more even contribution from each compartment, indicate lower efficiency in resource use within the system. These processes are quantified by OD and OE, respectively.

(5) O=OI+OE+OD+R

The responses of DC, *A*, and *O* may be mostly driven by TST when the magnitude of TST exceeds that of the structural components *H*, AMI, and Hc. To resolve this issue, we normalized ascendency and overhead by the upper limit (DC), yielding relA = *A*/DC and relO = *O*/DC. To clarify the role of the food web in shaping system's responses, without the confounding effect of imports, exports, and respiration, we computed indices based exclusively on intercompartmental exchanges. These indices are analogous to those calculated in Equations (4) and (5) and quantify the upper limit to internal growth (internal development capacity, IDC), the internal flow organization (internal ascendency, IA), and redundancy (IR), with a focus on the structural component of the latter (IHc). Weighted network connectance was quantified considering all the exchanges between the compartments, including both living and nonliving nodes (intercompartmental connectance, ICC), or using biotic compartments only (food web connectance, FWC). To understand whether any relationship exists between the internal flow structure and system's efficiency of resource usage, we calculated the average residence time (ART) of carbon in the system. ART is computed as the ratio between the total compartments' biomass and the sum of all flows leaving the system (i.e., respiration and exports).

#### Ecosystem attributes

Ecosystem attributes were calculated to interpret changes in ecological network structure (Appendix S1: Table S7). To investigate the importance of input, export, and respiration flows, we computed the total primary productivity by summing the photosynthesis of the three macrophytes (GPP); the total respiration, that is, sum of the energy lost from the system by macrophytes and consumers (Resp); and the total export, that is, sum of primary producers' biomass floating in the tanks (energy that leaves the system but is still usable outside of its boundaries, Export). Herbivory, detritivory, and detritivory to herbivory ratio (DH) were calculated to assess for the importance of alternative energy sources. Whether consumption depends on grazing or on dead organic matter, and how the relative importance of these feeding modes varies, may explain changes in connectance. Biomass‐related attributes help interpret how bottom‐up and top‐down forces interact to shape the ecosystem network. We then included in the analysis the total biomass of consumers (CB); total biomass of macrophytes (MB); and POC biomass. The sum of all consumers' flows to POC (dead organisms and feces; POCsink) was modeled to further the interpretation of system's connectance.

### Statistical and network analysis

Modeling was performed in R (ver. 4.5.0; R Core Team, 2025). Network analysis was conducted with ad hoc scripts. We applied generalized linear models (GLMs) with family distribution Gamma to identify changes in the network indices and ecosystem attributes using the *stats* package. Diagnostic plots were used to compare model performance under Gaussian and Gamma distributions, with Akaike information criterion (AIC) scores further supporting the choice of the Gamma family (with inverse link function), which more effectively accommodates skewed data. GLMs were applied to test the differences between the heat wave (1HW, 3HW) and control (0HW) treatments. Networks were visualized using *DiagrammeR* (Iannone & Roy, 2016) and data were plotted with *ggplot2* (Wickham, 2016).

## RESULTS

Tanks exposed only to the final summer heat wave (1HW) show a greater impact on the indices than those in the 3HW treatment. Both treatments boost detritivory, which causes the systems to increase their scope for IDC. Although certain df persist in carbon circulation (*R*), the overall ICC of 1HW trophic networks is lower than under control conditions, indicating the strengthening of interactions among consumers and POC (Figure 2). This finding is confirmed by the stronger magnitude of carbon flows towards POC (POCsink), which stands for a combination of increased consumers' egestion and mortality. 1HW systems process more carbon than control tanks (TST, difference not significant) but do so less efficiently because of higher respiration losses and a reduced capacity to retain carbon in the trophic network (ART).

### Information theory indices

**FIGURE 3:**
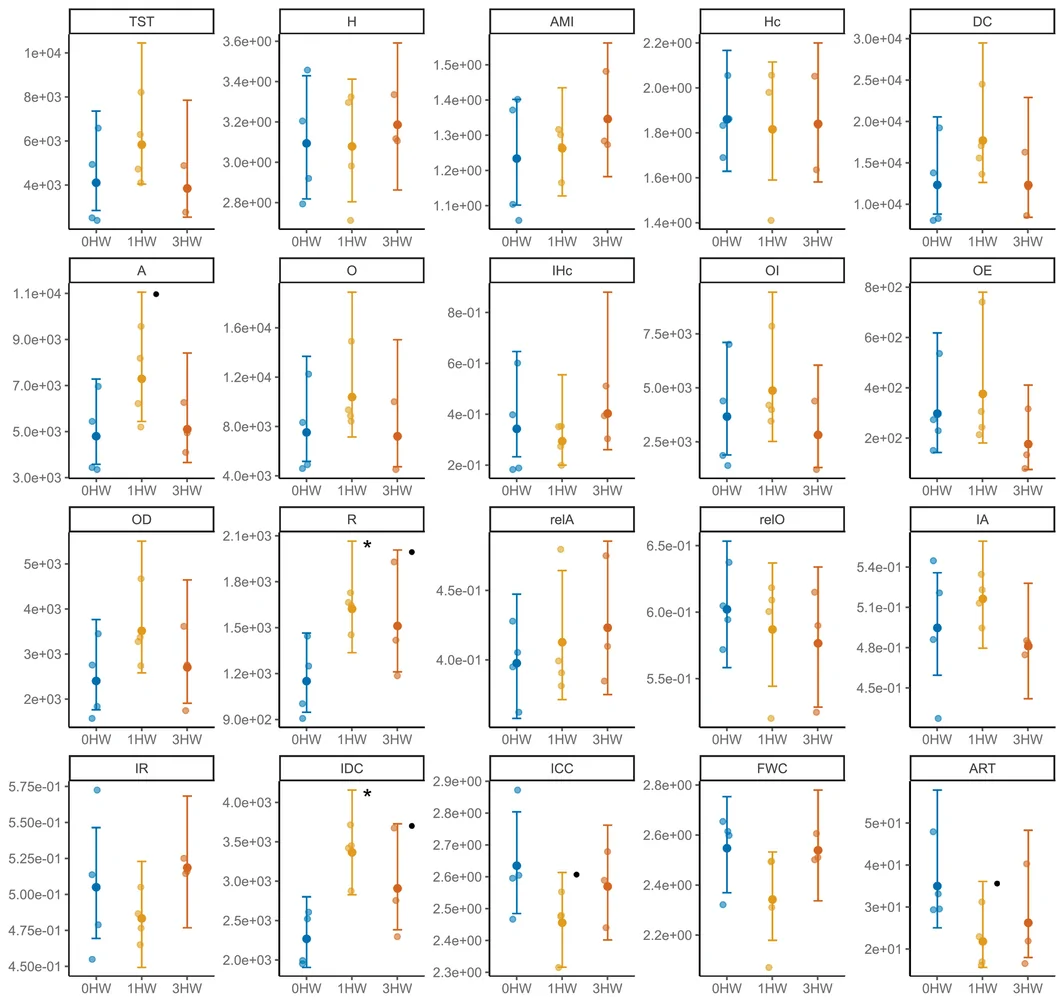
Network indices are shown as generalized linear model (GLM)‐fitted estimates (dark circles) with 95% CI represented by the error bars. The lighter‐color circles visualize the values calculated using the carbon flow networks built for each tank. Heat wave treatments: no heat waves (0HW) (blue), one late‐summer heat wave (1HW) (yellow), and three sequential heat waves spanning late spring–summer (3HW) (red). Flux‐based indices are reported in milligrams of carbon per square meter per day. Differences between 0HW and heat wave treatments (1HW and 3HW) are indicated by asterisks for significant effects (*p* < 0.05) and by black circles for marginally significant effects (0.05 ≤ *p* < 0.10). Indices: A, ascendency; AMI, average mutual information; ART, average (carbon) residence time (day); DC, development capacity; FWC, food web connectance; *H*, flow diversity; Hc, residual diversity; IA, internal ascendency; ICC, intercompartmental connectance; IDC, internal development capacity; IHc, internal residual diversity; IR, internal redundancy; O, overhead; OD, dissipative overhead; OE, overhead on exports; OI, overhead on imports; R, redundancy; relA, relative ascendency; relO, relative overhead; TST, total system throughput.

Although many indices varied little under 1HW and 3HW compared to 0HW, some clear trends emerged. The TST did not differ significantly between treatments (Appendix S2: Table S1), but a slight increase after 1HW was observed (Figure 3). None of the indices quantifying various facets of flow diversity (*H*, AMI, and Hc) responded and this lack of significance was reflected in the statistical tests of DC and *O*, despite increasing trends for both indices when calculated for systems exposed to 1HW in comparison to 0HW and 3HW (Appendix S2: Table S1). Although AMI did not differ between treatments, A displayed a marginally significant (estimate = −7.08 × 10^−5^ ± 3.67 × 10^−5^, *t*_2,8_ = −1.932, *p* = 0.089) increase after 1HW compared to 0HW. The overhead did not respond to heat waves, and indices OI, OE, and OD likewise showed no response. Nevertheless, *R* increased after exposure to 1HW (estimate = −2.52 × 10^−4^ ± 9.86 × 10^−5^, *t*_2,8_ = −2.552, *p* = 0.034) and 3HW (estimate = −2.06 × 10^−5^ ± 1.07 × 10^−4^, *t*_2,8_ = −1.926, *p* = 0.090). The normalized versions of *A* (relA) and *O* (relO) did not show significant differences across treatments, which indicates that the response found for *A* after 1HW was promoted by the TST. The indices IA and IR corroborate that the organization of internal flows, which excludes the flows crossing the system's boundaries, did not differ between the control and the heat wave treatments (Appendix S2: Table S1). However, IDC increased after 1HW (estimate = −1.43 × 10^−4^ ± 4.37 × 10^−5^, *t*_2,8_ = −3.275, *p* = 0.011) and 3HW (estimate = −9.66 × 10^−5^ ± 4.88 × 10^−5^, *t*_2,8_ = −1.98, *p* = 0.083) compared to 0HW (Appendix S2: Table S1). Despite the increase of IDC in both heat wave treatments, ICC displayed a marginally significant decrease in the ecosystems exposed to 1HW (estimate = 2.76 × 10^−2^ ± 1.45 × 10^−2^, *t*_2,8_ = 1.898, *p* = 0.094) but not in the presence of 3HW (estimate = 9.63 × 10^−3^ ± 1.54 × 10^−2^, *t*_2,8_ = 0.626, *p* = 0.548). Consistently, ART exhibited a marginally significant decline after 1HW (estimate = 1.71 × 10^−2^ ± 9.21 × 10^−3^, *t*_2,8_ = 1.861, *p* = 0.099) but not in response to 3HW (estimate = 9.46 × 10^−3^ ± 8.95 × 10^−3^, *t*_2,8_ = 1.057, *p* = 0.321), relative to 0HW. The decrease of ICC was driven primarily by exchanges with the nonliving compartment, a conclusion supported by the absence of a significant difference between FWC values after heat waves and FWC values in the 0HW treatment (1HW: estimate = 3.42 × 10^−2^ ± 1.88 × 10^−2^, *t*_2,8_ = 1.821, *p* = 0.106; 3HW: estimate = 1.23 × 10^−3^ ± 1.95 × 10^−2^, *t*_2,8_ = 0.063, *p* = 0.951; Appendix S2: Table S1).

Moreover, the Shannon diversity index, calculated based on compartment biomasses, was used in a linear model to test its relationship with flow diversity (*H*) and link‐weighted connectance (ICC). No significant relationships were found between the Shannon diversity index and either *H* (*F*_5,5_ = 0.288, *p* = 0.901) or ICC (*F*_5,5_ = 1.468, *p* = 0.342). These results indicate no direct relationship between state variables (biodiversity) and system processes (carbon exchanges), even though the latter depend on compartment biomasses for their quantification.

### Ecosystem attributes

**FIGURE 4:**
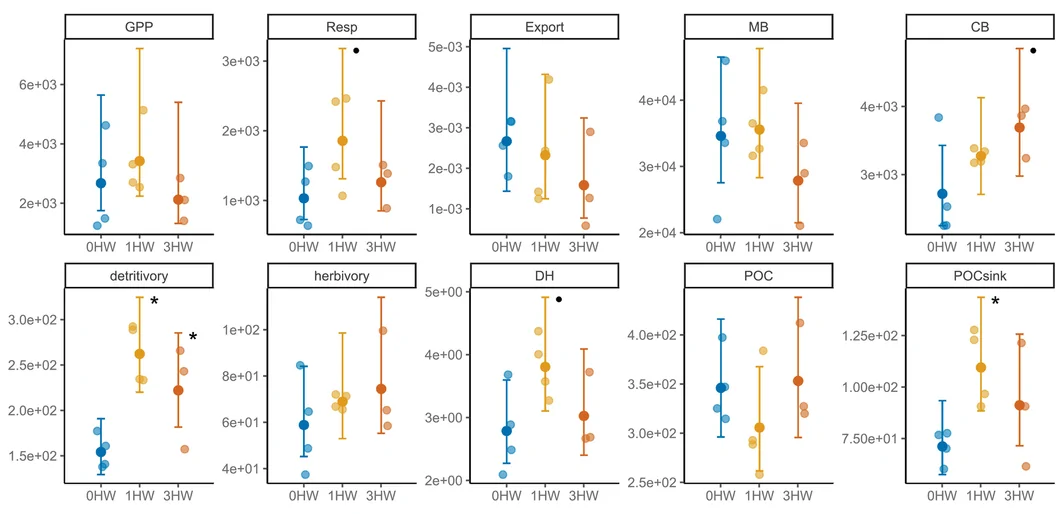
The ecosystem attributes are shown as generalized linear model (GLM)‐fitted estimates (dark circles) with 95% CI represented by the error bars. The lighter‐color circles visualize the values calculated using the carbon flow networks built for each tank. Heat wave treatments: no heat waves (0HW) (blue), one late‐summer heat wave (1HW) (yellow), and three sequential heat waves spanning late spring–summer (3HW) (red). Flux‐based indices are reported in milligrams of carbon per square meter per day; standing stock‐based indices in milligrams of carbon per square meter. Differences between 0HW and warming treatments (1HW and 3HW) are indicated by asterisks for significant effects (*p* < 0.05) and by black circles for marginally significant effects (0.05 ≤ *p* < 0.10). Attributes: CB, consumers' biomass; detritivory; herbivory; DH, detritivory to herbivory ratio; Export, exports from primary producers; GPP, total gross primary production; MB, primary producers' biomass; POC, particulate organic carbon; POCsink, amount of carbon that sinks to POC; Resp, total respiration.

GPP and exports, two attributes associated with macrophytes, did not differ between treatments (Figure 4, Appendix S2: Table S2). Nevertheless, the total respiration in the 1HW treatment increased compared to 0HW (estimate = −4.30 × 10^−4^ ± 2.00 × 10^−4^, *t*_2,8_ = −2.152, *p* = 0.063). These physiological responses can only be partly explained by changes in biomass as MB did not differ between treatments while CB marginally increased, compared to 0HW, after exposure to 3HW (estimate = −9.66 × 10^−5^ ± 4.33 × 10^−5^, *t*_2,8_ = −2.232, *p* = 0.056). Detritivory exhibited a pronounced increase in both heat wave treatments relative to 0HW (1HW: estimate = −2.66 × 10^−3^ ± 6.25 × 10^−4^, *t*_2,8_ = −4.261, *p* = 0.003; 3HW: estimate = −1.97 × 10^−3^ ± 6.91 × 10^−4^, *t*_2,8_ = −2.859, *p* = 0.021) while herbivory did not change after either heat wave treatments. Under 1HW, the increased detritivory was sufficient to cause a marginally significant rise in the DH ratio relative to the control (estimate = −9.58 × 10^−2^ ± 4.33 × 10^−2^, *t*_2,8_ = −2.21, *p* = 0.058), whereas no significant change was detected after 3HW (estimate = −2.83 × 10^−2^ ± 5.10 × 10^−2^, *t*_2,8_ = −0.553, *p* = 0.595). The growing relevance of the nonliving component under 1HW and 3HW was not reflected by the POC biomass, which did not show any significant differences between 0HW and the heat wave treatments. The extent of egestion and natural mortality causing carbon flows from consumers to POC (POCsink) increased instead after 1HW in comparison to 0HW (estimate = −4.90 × 10^−3^ ± 1.72 × 10^−3^, *t*_2,8_ = −2.884, *p* = 0.022).

## DISCUSSION

Exposure to either single or sequential heat waves modifies species' abundance and biomass (Pansch et al., 2018), their physiology, and ecological interactions (Ito et al., 2024). In this work, we found that impacts on species physiology, biomass, and trophic interactions cascade upward through the ecological hierarchy, reshaping the structure of ecosystem carbon fluxes and, consequently, the value of flow‐based indices derived from information theory. We show that network indices detect even subtle changes triggered by sublethal heat waves. Major responses were observed in the 1HW tanks, suggesting that experiencing sequential events within the species' thermal tolerance ranges promote ecosystem acclimation. Information theory indices provide a robust framework for linking network structure to aspects of ecosystem functioning like energy transfer efficiency and food web stability (Rutledge et al., 1976; Scotti et al., 2022). However, existing works were based on either the simulation of shortgrass prairie ecosystem succession (Rutledge et al., 1976) or trophic networks constructed by integrating biomasses from long‐term surveys with metabolic rates and feeding preferences derived from the literature (e.g., Scotti et al., 2022).

This study is the first to quantify metabolic rates, biomasses, and feeding preferences using an experiment tailored to assess ecosystem responses to an anthropogenic stress factor, that is, heat waves. It demonstrates that ecosystems respond to disturbance by reducing the diversity of carbon flows, thereby compromising stability and resilience (Ulanowicz, 2000). The increased fragility displayed by ecosystems impacted by heat waves transcends the direct effects on metabolism (e.g., respiration stress exhibited by the gastropod *L. littorea* under 1HW and egestion flows; POCsink in Figure 4), cannot be explained by biomass distribution (i.e., nonsignificant relationships linking Shannon diversity index of biomasses to flow diversity and ICC; see also Ulanowicz, 2018), and partly depends on dietary shifts towards detritivory. Ecosystem fragility, driven by the dominance of a few interactions controlling carbon circulation, stems from responses across different levels of the ecological hierarchy (Ito et al., 2024). These findings indicate that, beyond posing a direct threat to organisms, as shown by the increased community respiration rates (Figure 4), heat waves also amplify the ecosystem's vulnerability to additional disturbances. Our results highlight that information theory indices respond to stress and aid in interpreting the transformations undergone by the ecosystem following heat wave exposure (Figure 3). These indices rest upon strong theoretical foundations because they are algebraic transformations of joint and marginal probability distributions derived from normalized flow matrices (Ulanowicz, 1986, 2004). This toolkit of indices is based on trophic interaction diversity (*H*; see MacArthur, 1955), which is partitioned into two complementary and mutually dependent terms: AMI and residual diversity (Hc) (Rutledge et al., 1976). A change in one produces an opposing variation in the other, resulting in what Ulanowicz (2025) describes as a Hegelian dialectic. A reduction in the constraints on carbon circulation, quantified by AMI, is accompanied by a concomitant increase in the freedom associated with carbon fluxes (Hc). This relationship reflects the dual nature of ecosystems, in which the entropic component of *H*, represented by Hc, enables trophic networks under ambient conditions (0HW) to respond to disturbance (i.e., heat waves). These df allow carbon to be rerouted through preferential pathways (i.e., detritivory). Exposure to heat waves increases constraints along these pathways, a shift that is captured by observed trends of AMI. Considering that the system was disturbed at sublethal levels, the directional changes displayed by information theory indices based on interactions responded coherently to the disturbance (Appendix S1: Table S6), thus exhibiting desirable properties for management‐relevant indices (Layton et al., 2016; Otto et al., 2018; Rombouts et al., 2013; Tam et al., 2017).

### Ecological significance of the indices

**FIGURE 5:**
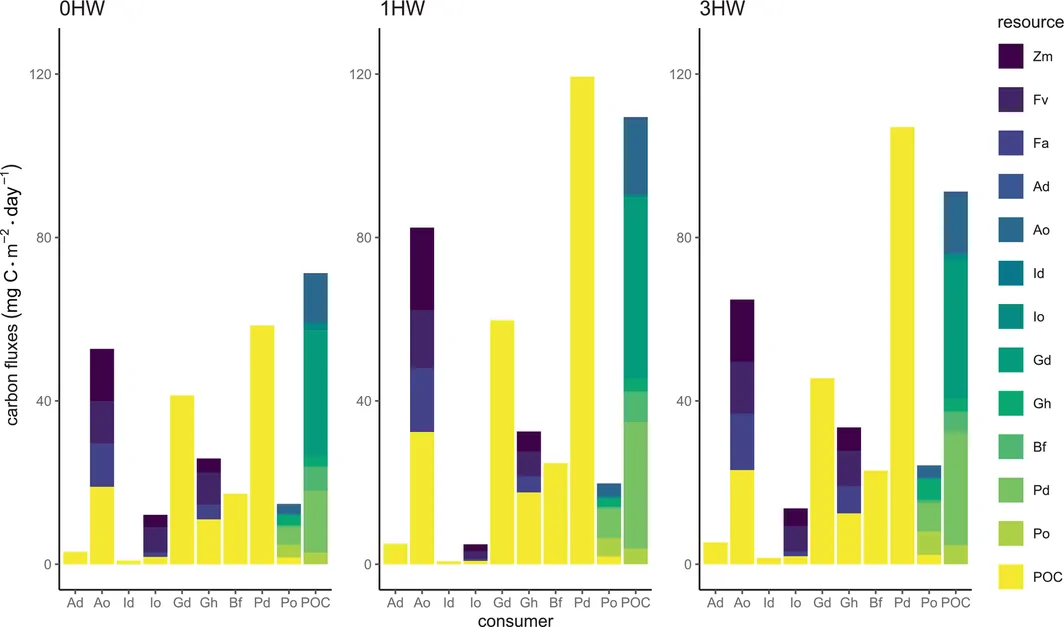
Total consumption by each compartment, apportioned among its resources. The bar plot illustrates that particulate organic carbon (POC) is a major carbon source for consumers, most of which increased their consumption in response to heat waves. 0HW, no heat waves; 1HW, one late‐summer heat wave; 3HW, three sequential heat waves spanning late spring–summer; Ad, amphipods detritivores; Ao, amphipods omnivores; Bf, bivalves filter‐feeders; Gd, gastropods detritivores; Gh, gastropods omnivores; Fa, filamentous algae; Fv, *Fucus vesiculosus*; Id, isopods detritivores; Io, isopods omnivores; Pd, polychaetes detritivores; Po, polychaetes omnivores; Zm, *Zostera marina*.

Heat waves alter feeding preferences and consumption rates (Ito et al., 2024), driving a shift towards detritivory (Figure 4). Increased consumption by amphipods omnivores, gastropods detritivores, bivalves filter‐feeders, and polychaetes detritivores was observed in both heat wave treatments compared to 0HW (Figure 5). It may have been caused by warming enhancing consumers' metabolic activity (Ito et al., 2019) and could explain the intensification of detritivory. Concomitantly, more carbon was egested from consumers towards detritus (POCsink, significant after 1HW), reducing the relative importance of carbon circulating through the grazing chain despite some increases in the magnitude of herbivory.

The prevalence of detritus‐related interactions under 1HW is coherent with the rise of ascendency and the decline of intercompartmental connectance (Figure 3). Consumers may have shifted towards feeding on POC as it offers higher digestibility compared to seagrasses and bladderwrack (Campanyà‐Llovet et al., 2017). Moreover, detritivory may benefit heterotrophs through the ingestion of bacteria, which supply energy and specific nutrients (Gontikaki et al., 2011; Kelly & Scheibling, 2012). Elevated egestion by consumers may have in turn stimulated a short‐term increase in bacterial production and metabolic activity (Grenz et al., 1990), thereby triggering a feedback response that indirectly reinforced consumers' reliance on POC. Exposure to the final summer heat wave likely accelerated consumers' metabolic responses, a hypothesis corroborated by a significant increase in respiration (Figure 4). The main consequence of this sustained cumulative dissipation is the contraction of carbon ART. The trends exhibited by total respiration and ART describe ecosystems exposed to 1HW as poorly effective in utilizing carbon and this inefficiency translates into stronger dissipation losses. Previous works showed community respiration to increase under heat waves (Göbeler et al., 2025). Although Göbeler et al. (2025) found that heat waves had negative influence on polychaetes detritivore adults (*Marenzelleria* sp.), in our experiment they thrived and their total biomass was higher under heat wave treatments than in the control (Ito et al., 2024). High biomasses of Pd led to higher consumption (Figure 5) and respiration rates than under 0HW. Not all consumers showed the capacity to increase their consumption to cope with a single heat wave at the end of summer. The common periwinkle (*L. littorea*) showed signs of stress following 1HW, with a significant increase in its respiration rates (Ito et al., 2024). This was accompanied by a decline in the biomass of omnivorous gastropods, but no detectable change in their total consumption (Figure 5). Although 1HW negatively affected the biomass of gastropods omnivores, IDC and redundancy increased, exhibiting a pattern that appears contradictory to the concurrent rise in A and ICC (Figure 3). Despite the lack of statistical significance, TST and AMI together determine the increase in A, as they show consistent trends distinguishing 1HW from 0HW. The strength of intercompartmental flows (Figure 5) was decisive to cause IDC and R to be higher under 1HW compared to 0HW. Analogously, the rise of A matches the higher values of respiration, detritivory, and egestion (POCsink) found in 1HW tanks compared to the control (Figure 4). These attributes indicate that in the 1HW ecosystems: (1) more energy was dispersed through respiration and (2) the simplification of the network relates to the dominance of flows composing the detritus food chain.

Exposure to three sequential heat waves increased IDC, *R*, and detritivory (Figures 3 and 4). However, 3HW led to an increase in consumers' biomass, accompanied by a nonsignificant decline in macrophytes' biomass (Figure 4). Herbivory did not vary significantly although grazing over macroalgae increased when the ecosystem was exposed to 3HW (Figure 5). The concurrent rise of detritivory and herbivory under 3HW explains why DH ratios remained comparable to those in the 0HW treatment. Despite the top‐down control exerted by mesograzers on macrophytes (particularly, filamentous algae; Ito et al., 2024) after 3HW, the indices show that the ecosystems became more resilient compared to those under 0HW (Figure 3). The decline of macrophytes was not clearly detected by the indices due to the generalist feeding of consumers. Generalist consumers may act as hubs by transmitting disturbances across the trophic network and triggering indirect effects of stressors throughout the ecosystem (Martins et al., 2024). Top‐down control imposed by mesograzers on primary producers may have been mitigated by their generalist feeding habits, which promoted redundancy in carbon flows (Guimaraes, 2020). Our results show that sequential, sublethal heat waves supported generalist feeding, thereby enhancing the diversity of carbon flows. This contrasts with cases where heat waves exhibited lethal effects (Garrabou et al., 2022), disrupting the functioning of entire marine ecosystems (Guibourd de Luzinais et al., 2024). Conversely, simulations run with an end‐to‐end ecosystem model found a slight increase in the complexity of trophic interactions after exposure to heat waves (Gomes et al., 2024). They observed that connectance (the ratio of existing links to the squared number of compartments) and link density (the average number of links per compartment) do not change substantially, indicating that MHWs do not reduce food web robustness to disturbance. Our analysis shows that a single, sublethal heat wave (1HW) disrupts connectance, while ecosystems previously exposed to sequential heat waves (3HW) did not display the same response. Many studies revealed that the functioning of ecosystems could bounce back from pulse disturbances (Hillebrand & Kunze, 2020). In freshwater experimental ecosystems, long and sequential heat wave effects on connectance may differ (Polazzo et al., 2023). At the end of the recovery period, the authors found that link‐weighted connectance (LWC) did not change after sequential heat waves but increased following exposure to a single, prolonged heat wave. Our experiment yielded comparable results as no changes in LWC occurred when the system was subjected to three sequential, sublethal heat waves.

The experimental dimension of the study could potentially represent a limit. For instance, no higher trophic levels were included in this experiment, which could alter system's dynamics. Nevertheless, trends observed in this mesocosm communities hold in large‐scale natural ecosystems, where disturbance has been shown to reduce the diversity of flows, resulting in more constrained pathways pertaining matter circulation. This pattern has been observed both when focusing on the architecture of trophic interactions disturbed by warming (e.g., the Crystal River ecosystem after exposure to thermal pollution caused by the discharge of heated cooling water from the Crystal River Energy Complex; Ulanowicz, 1996) and when simulating changes in ecosystem structure and carbon flow strength in response to varying intensities of fishing mortality (i.e., fishery scenarios developed using the OSMOSE model of the Eastern English Channel; Ito et al., 2023). Our study confirms that ecosystems preserve higher degrees of unimpeded matter circulation under undisturbed conditions (Scotti et al., 2022).

### Management significance of the indices

Maintaining a focus on ecological interactions is fundamental to the implementation of ecosystem‐based management (EBM) strategies (Rocchi et al., 2017; Tam et al., 2017). EBM is often supported by integrated ecosystem assessment, an iterative process where indicators are (1) developed to ensure the achievement of management objectives, and (2) applied to monitor ecosystem status and assess the effectiveness of management actions in meeting the initial goals (Levin et al., 2009). It is therefore necessary to identify holistic indicators that capture changes in link arrangement and the strength of ecological interactions in response to anthropogenic disturbances (Rombouts et al., 2013). These indicators must also be grounded in robust theoretical foundations (Otto et al., 2018). Our work relies on mesocosms to test whether interaction‐based indices derived from information theory are responsive to heat waves. It focuses on the structure and magnitude of carbon fluxes to show that several holistic indices respond to heat waves. The most pronounced changes occurred in ecosystems exposed to 1HW, suggesting that either ecological stress memory (Ito et al., 2024) or the interplay between synergistic and antagonistic forces across the ecological hierarchy may have contributed to the attenuated responses observed under 3HW.

To date, modeling studies have demonstrated the validity of information theory indices for tracking the impacts of perturbations targeting specific trophic groups, such as fisheries (Ito et al., 2023), and for examining ecosystem responses to disturbance in simulations based on an evolutionary ecology theoretical framework (Brinck & Jensen, 2017). While some experiments have explored the responses of trophic interactions to perturbations (Polazzo et al., 2022, 2023), none have assessed the suitability of the information theory toolkit from ENA. Here, we provide empirical evidence supporting the utility of these indices to detect ecosystem responses, even with warming not exceeding species' physiological tolerance. Comparisons with ecological attributes help to interpret the changes quantified by network indices. Ecosystems responded to heat waves by increasing detritivory, while reducing their efficiency of carbon usage (higher respiration losses) and strengthening their connection with detritus (increased egestion from consumers) after 1HW. Explaining the responses of network indices through ecological attributes reduces the risk of these metrics being perceived as black box tools lacking ecological relevance. Furthermore, the use of an experimental setting helps clarify the sensitivity of the indices to warming. The robustness of these indices is reflected in their ability to capture the influence of heat waves on the overall ecosystem, despite trophic groups' responses spanning from physiological changes to shifts in feeding (Ito et al., 2024). This capacity to provide an integrated assessment of dynamic responses to anthropogenic pressures, including the detection of direct and indirect effects, represents a desirable feature of food web indicators (Tam et al., 2017). Our results support the use of information theory indices to reveal shifts in carbon flow structure. They confirm that flow diversity and internal carbon usage increase under undisturbed conditions while reduced complexity and higher losses prevail in perturbed ecosystems (Ito et al., 2023; Rutledge et al., 1976; Scotti et al., 2022). This level of uniformity and consistency in the responses is not always present. For example, the amount of carbon reused in the network, often quantified by the Finn Cycling Index, is expected to increase as ecosystems progress towards maturity; however, a similar trend may also be found under stress caused by excessive nutrient availability, even though in this latter case most of the cycling occurs via short routes (Patrıcio et al., 2004). Their sensitivity to stress and coherence in response direction make information theory indices well suited to support managers aiming to implement legislation aligned with EBM.

In the European Union (EU), there is the urgent need for indicators capturing changes in ecosystem structure and functioning to ensure an effective transition towards EBM (Safi et al., 2019). The EU Water Framework Directive (WFD, 2000) implicitly asks for the protection of aquatic ecosystems by defining the ecological status as “an expression of the quality of the structure and functioning of aquatic ecosystems.” Structural and functional properties can be modeled by information theory indices that provide a numeric assessment of the ecological status. Our findings demonstrate that these indices offer a robust framework, as they integrate the contributions of multiple biological components. They capture responses across organizational levels, from physiology (e.g., respiration rates) and population dynamics (e.g., biomass changes) to shifts in feeding preferences quantified via SIA. The EU Common Fisheries Policy (CFP, 2013) calls for an ecosystem‐based approach to fisheries management and information theory indices may quantify ecosystem‐level impacts of fishing. They may be calculated to monitor how alternative fisheries management plans contribute to protecting marine ecosystems. The EU MSFD (2008, 2017) demands applying EBM to achieve the “sustainable use of marine goods and services” and requires that “priority should be given to achieving or maintaining good environmental status in the Community's marine environment.” The MSFD defines nine descriptors to determine whether marine ecosystems achieve GES; descriptor D4 deals with food web assessment and implies that indicators integrating responses across multiple trophic levels and considering processes and linkages among trophic groups should be applied (Safi et al., 2019). Presently, most of the indicators used by the Convention for the Protection of the Marine Environment of the North‐East Atlantic (OSPAR) and the Baltic Marine Environment Protection Commission (HELCOM) to assess the state of marine food webs focus on trophic guilds (e.g., Thompson et al., 2020) while ENA‐derived indices have been only proposed as candidates (HELCOM, 2023, pp. 210–214; Schückel et al., 2022). Here, we present experimental evidence on the reliability and sensitivity of information theory indices in characterizing food web responses to anthropogenic disturbance. Our results, supported by the ecological theory underlying the indices, indicate their relevance for addressing MSFD criteria D4C2 and D4C4. We demonstrated that information theory indices capture imbalances in the abundance of trophic guilds (e.g., CB after 3HW) and respond to changes in productivity and increases in respiration. Additionally, they detect system‐level responses preceding major changes in trophic groups, for example through the decline of LWC and the rise of ascendency following 1HW.

The applicability of information theory indices extends beyond EU boundaries. These indices could be used to promote evidence‐based biodiversity management as demanded by target 21 of the Kunming‐Montreal Global Biodiversity Framework (https://www.cbd.int/gbf/targets/notes.shtml). They could track the consequences of fisheries and operationalize the Ecosystem Approach to Fisheries (EAF) adopted by the FAO Committee on Fisheries. EAF encourages in fact the monitoring of trophic interactions and ecosystem structure to guide fisheries management (Garcia et al., 2003), both of which are captured by information theory indices. These indices may help achieve the goals of the Intergovernmental Science‐Policy Platform on Biodiversity and Ecosystem Services, which recognizes the critical role of trophic interactions in ecosystem functioning (IPBES, 2016, pp. 125–127).

## CONCLUSIONS

Discrepancies between ecosystem attributes and indices based on information theory highlight the need to consider properties derived from processes (trophic interactions) when assessing ecosystem status (Ulanowicz, 2018). Data generated from mesocosms have already been used to support real‐world model construction and scenario development (Ullah et al., 2024). Here, we demonstrate that experimental settings are appropriate for testing the responsiveness of indices to disturbance and for disentangling the mechanisms underlying changes in these metrics. Empirical evidence from our study, combined with previous modeling efforts and established theoretical foundations, supports the use of information theory indices as a robust toolkit for assessing the status of marine food webs.

## AUTHOR CONTRIBUTIONS

**Maysa Ito:** Conception and design; data acquisition; analysis and interpretation of results; drafting of the article; preparation of the final manuscript version. **Anna Waffender:** Data analysis and critical revision of the article. **Marco Scotti:** Conception and design; data acquisition; analysis and interpretation of results; drafting of the article; preparation of the final manuscript version.

## CONFLICT OF INTEREST STATEMENT

The authors declare no conflicts of interest.

## Supporting information


Appendix S1.

Appendix S2.

## Data Availability

Data are available in PANGAEA in the following data releases: Ito, Weinberger, et al. (2024), https://doi.org/10.1594/PANGAEA.966174; Ito and Scotti (2024a), https://doi.org/10.1594/PANGAEA.966180; Ito and Scotti (2024b), https://doi.org/10.1594/PANGAEA.966179.
