# Supplementary material for: Network indices show heat wave‐induced fragility in experimental food webs: Operational insights for ecosystem management
Source: Ecol Appl. 2026 Sep 8;36(6):e70277. doi: 10.1002/eap.70277 (PMC13554289; doi:10.1002/eap.70277)
Supplement: Supplementary file 1 [file EAP-36-e70277-s001.pdf]

Network indices show heat wave-induced fragility in experimental food webs: Operational insights for ecosystem management

Maysa Ito, Anna Waffender, and Marco Scotti

Ecological Applications

Appendix S1: Methods

Section S1: Biomass quantification

The wet mass (WM) of seagrasses and bladderwracks was estimated using monthly growth rates. The WM was converted to dry mass (DM) based on Kraemer & Alberte (1993) and Küppers & Kremer (1978) for *Zostera marina* and *Fucus vesiculosus*, respectively. The biomass of the filamentous algae *Ceramium* spp. was obtained through direct measurement of DM. The biomasses of *Idotea balthica*, *Gammarus* spp. and *Littorina littorea* were inferred using length-dry mass regressions (LMRs) built from individuals collected during the experiment (see Ito *et al.*, 2024). DM biomasses of amphipods detritivores, isopods detritivores, gastropods detritivores, bivalves filter-feeders, polychaetes omnivores, and polychaetes detritivores were obtained by weighting all individuals collected at the end of the experiment, following oven-drying at 80 °C for at least 48 hours. In the case of amphipods, isopods and gastropods detritivores, and bivalves filter-feeders, oven-dried biomasses were summed with those estimated from literature-derived LMRs (Rumohr *et al*., 1987), which were applied to a subset of individuals sampled before ending the experiment. For all compartments, DM was converted to carbon using carbon content values measured concurrently with the stable isotope analysis for each treatment. To quantify particulate organic carbon (POC), from each mesocosm tank, 180 ml of seawater was filtered through Whatman GF/F glass microfiber filters (pore size 0.7 µm). The filters were dried at 60 °C for at least 48 hours and the carbon content was determined by gas chromatography using an elemental analyzer system (Thermo Flash, 2000; Thermo Fisher Scientific Inc., Schwerte, Germany). The POC content of each mesocosm unit was normalized by the filtered volume and estimated for the whole tank by multiplying the value by the total volume of the tank (1,500 L). Biomasses were expressed in mg C m^{-2} and calculated by dividing the total carbon content of each compartment by the base area of the tank (1.53 m^2).

Section S2: Quantification of primary production, respiration, and exports of macrophytes

The net primary production (NPP) and respiration rates of seagrasses, bladderwracks and filamentous algae were measured between 10 and 14 August 2015 across all treatments. The macrophytes were incubated in 6 L gas-tight clear acrylic cylindrical chambers equipped with a stirrer and a non-invasive oxygen sensor spot PSt3 (PreSens Precision Sensing GmbH, Regensburg, Germany). The sensor spots were read using the fiber-optic oxygen meter Fibox 4 (PreSens Precision Sensing GmbH, Regensburg, Germany), allowing the recording of dissolved oxygen concentration in sealed chambers after either one (*F. vesiculosus* and filamentous algae) or four hours (*Z. marina*) of incubation. To maintain the temperature at the same level as in the tanks, the incubation chambers were maintained fully submerged with top lid at ca. 10 cm depth. NPP incubations were performed under natural light conditions, outdoor in the mesocosms), while respiration measurements were executed in the dark by placing a black, light-sealed cover over the chambers. Along each incubation, a reference chamber containing either only non-filtered seawater (*F. vesiculosus* and filamentous algae) or non-filtered seawater and sediment in a beaker (*Z. marina*) was used to correct changes in oxygen concentration unrelated to the macrophytes (i.e., planktonic and bacterial activity). Oxygen values were converted to daily carbon rates accounting for changes measured (in mg O_2 L^{-1} h^{-1}), volume of the chamber (in L), photosynthesis and respiration quotients (i.e., depending on the organism), molar masses of C and O_2, and biomass of incubated specimens (mg C).

The exports were quantified for the macrophytes. We considered as exports the floating material in the tanks that was detached from seagrasses and macroalgae. In the absence of tank walls, this material would have been transported beyond the ecosystem's boundaries by water currents thus representing a source of usable energy. Once per week, we collected leaves and thalli of primary producers (*Z. marina*, *F. vesiculosus*, and filamentous algae) found detached and floating in the tanks. The material was separated and dried at 80 °C for at least 48 hours, and the biomass was then quantified.

Section S3: Respiration rates of consumers

The respiration incubations were performed in the laboratory using circulating water baths (Thermo Fisher Scientific, Darmstadt, Germany) to maintain the temperature at the same levels as in the Kiel Outdoor Benthocosm. Mesograzers were enclosed in 100 mL Winkler bottles containing filtered seawater (0.2 µm Whatman mixed cellulose ester filter; GE Healthcare Life Sciences, Germany) and the oxygen consumption was logged for about one hour using the

Multi-channel Fiber Optic Oxygen Meter Oxy-10 mini (PreSens Precision Sensing GmbH, Regensburg, Germany). Control incubations of filtered seawater were carried out to detect oxygen concentration changes due to reasons unrelated to the respiration of sampled organisms (e.g., bacterial activity). Respiration values were converted from mg O_2 L^{-1} h^{-1} to mg C m^{-2} day^{-1} using a respiration quotient of 0.85. Rates were calculated by dividing the respiration values by the biomass of the incubated individuals (mg C).

Section S4: Stable isotope analysis

The stable isotopes were obtained from samples of *Z. marina*, *F. vesiculosus* and filamentous algae, and individuals of *I. balthica*, *Gammarus* spp. and *L. littorea* collected from each experimental unit. The samples were washed with distilled water and frozen at -80 °C until processing. Then, they were dried at 60 °C for at least 48 hours. Dried samples were homogenised with agate mortar and pestle and kept in glass vials. The material was subsampled, weighed, and placed into tin capsules (3.2 × 4.0 mm, Hekatech, Wegberg, Germany). Samples were analysed with simultaneous measurements of $\delta^{13}C$, $\delta^{15}N$ and $\delta^{34}S$ using an elemental analyser system (NA 1110, Thermo, Milan, Italy) connected to a temperature-controlled gas chromatography oven (SRI 9300, SRI Instruments, Torrance, CA, USA) and to an isotope-ratio mass spectrometer (Delta^{Plus} Advantage, Thermo Fisher Scientific) as described in Hansen *et al.* (2009). Isotope ratios are expressed in δ values, according to equation S1.

$$\delta X = \left(\frac{R_{sample}}{R_{standard}} - 1\right) \cdot 1000 \qquad \text{(eqn. S1)}$$

Where δX represents the deviation in per mill (‰) from the standards (i.e., N_2, CO_2, and SO_2 that were used as reference gases) and R represents either the $^{13}C/^{12}C$, $^{15}N/^{14}N$ or the $^{34}S/^{32}S$ ratios. We used MixSIAR (Stock *et al.*, 2018) to model the stable isotope data and obtain relative contributions of resources to each consumer. The treatment was used as fixed factor and the enrichment factors were based on literature, i.e., 0.5‰ for $\delta^{13}C$, 2.4‰ for $\delta^{15}N$ and 0‰ for $\delta^{34}S$.

Section S5: Theoretical expectations for information theory indices

Systems under stress reduce their size (TST) and show a growing dominance of a few main pathways responsible for carbon circulation. These structural variations are reflected by decreases in Hc, O, or relO while AMI, A, or relA increase under stress; the changes expected for all indices in response to disturbance are summarized in Table S6. Previous works have

shown that both overhead and residual diversity increase across time in undisturbed ecosystems (e.g., Scotti *et al.*, 2022) while simulation experiments demonstrated a progressive loss of flow diversity with growing intensity of disturbance (e.g., Ito *et al.*, 2023). Our primary hypothesis is that heat waves increase the fragility of the trophic networks compared to ambient conditions (0HW). We expect responses of the 1HW treatment to be stronger than in the 3HW tanks where ecological stress memory may buffer the negative influence of sequential warming (Ito *et al.*, 2024).

Table S1. List of network compartments, corresponding codes, and associated species. Species were grouped in the same compartment according to similarities in trophic habits and habitat preferences. Particulate organic carbon represents detritus and is the only non-living compartment.

**Compartment**	**Code**	**Species**
Seagrasses	Zm	*Zostera marina*
Bladderwracks	Fv	*Fucus vesiculosus*
Filamentous algae	Fa	*Ceramium* spp.
Isopods omnivores	Io	*Idotea balthica*
Amphipods omnivores	Ao	*Gammarus spp.*
Gastropods omnivores	Gh	*Littorina littorea*
Isopods detritivores	Id	*Jaera albifrons*
Amphipods detritivores	Ad	*Microdeutopus* sp. and *Corophium* sp.
Gastropods detritivores	Gd	*Hydrobia* sp.
Bivalves filter-feeders	Bf	*Mytilus edulis*, *Macoma* sp. and *Mya truncata*
Polychaetes omnivores	Po	*Hediste* sp., *Scoloplos armiger* and *Phyllodoce mucosa*
Polychaetes detritivores	Pd	*Marenzelleria viridis*, *Polydora cornut*a and *Pygospio elegans*
Particulate organic carbon	POC	

Table S2. Production to biomass (P/B) ratios and assimilation efficiencies (α) extracted from the literature and used to quantify compartments' consumption (Q) for the heterotrophic organisms. The ratios were scaled with Q10 values to reflect the temperatures at which control (0HW) and heat wave (1HW, 3HW) treatments were exposed (20° C and 25°C, respectively). Assimilation efficiency too was specific to each treatment temperature. Literature sources consulted for each parameter are summarised. Ratios are expressed as day^{-1} and multiplying them by the biomass ($mg\ C\ m^{-2}$) allows obtaining production (P) flows ($mg\ C\ m^{-2}\ day^{-1}$) while α is dimensionless and when used to divide the gross production (GP = P + R) enables the calculation of the total consumption (Q = GP/α). When a value for a species was unavailable, the value from a similar taxon was used; the proxies employed are indicated in the reference column.

**Code**	**Species**	**Treatment**	**P/B (day^{-1})**	**References**	**α**	**References**
Io	*Idotea balthica*	0HW	0.0328	Redant (1989); Baden & Phil (1984)	0.8488	Ito *et al.* (2019)
Io	*Idotea balthica*	1HW	0.0658	Redant (1989); Baden & Phil (1984)	0.8973	Ito *et al.* (2019)
Io	*Idotea balthica*	3HW	0.0658	Redant (1989); Baden & Phil (1984)	0.8973	Ito *et al.* (2019)
Ao	*Gammarus* spp.	0HW	0.0117	Baird *et al.* (2004)	0.7778	Baird *et al.* (2004)
Ao	*Gammarus* spp.	1HW	0.0181	Baird *et al.* (2004)	0.7778	Baird *et al.* (2004)
Ao	*Gammarus* spp.	3HW	0.0181	Baird *et al.* (2004)	0.7778	Baird *et al.* (2004)
Gh	*Littorina littorea*	0HW	0.0154	Baird *et al.* (2004)	0.9043	Grahame *et al.* (1973)
Gh	*Littorina littorea*	1HW	0.0165	Baird *et al.* (2004)	0.9043	Grahame *et al.* (1973)
Gh	*Littorina littorea*	3HW	0.0165	Baird *et al.* (2004)	0.9043	Grahame *et al.* (1973)
Id	*Jaera albifrons*	0HW	0.0328	Redant (1989); Baden & Phil (1984)	0.8488	Ito *et al.* (2019); see *I. balthica*
Id	*Jaera albifrons*	1HW	0.0658	Redant (1989); Baden & Phil (1984)	0.8973	Ito *et al.* (2019); see *I. balthica*
Id	*Jaera albifrons*	3HW	0.0658	Redant (1989); Baden & Phil (1984)	0.8973	Ito *et al.* (2019); see *I. balthica*
Ad	*Corophium volutator*	0HW	0.0109	Baird *et al.* (2004)	0.8675	Tab. 2 in Nielsen & Kofoed (1982)
Ad	*Corophium volutator*	1HW	0.0169	Baird *et al.* (2004)	0.8675	Tab. 2 in Nielsen & Kofoed (1982)
Ad	*Corophium volutator*	3HW	0.0169	Baird *et al.* (2004)	0.8675	Tab. 2 in Nielsen & Kofoed (1982)
Ad	*Microdeutopus* sp.	0HW	0.0109	Baird *et al.* (2004); see *C. volutator*	0.8675	Tab. 2 in Nielsen & Kofoed (1982); see *C. volutator*
Ad	*Microdeutopus* sp.	1HW	0.0169	Baird *et al.* (2004); see *C. volutator*	0.8675	Tab. 2 in Nielsen & Kofoed (1982); see *C. volutator*

Ad	*Microdeutopus* sp.	3HW	0.0169	Baird *et al.* (2004); see *C. volutator*	0.8675	Tab. 2 in Nielsen & Kofoed (1982); see *C. volutator*
Gd	*Hydrobia ulvae*	0HW	0.0458	Baird *et al.* (2004)	0.2542	Baird *et al.* (2004)
Gd	*Hydrobia ulvae*	1HW	0.0602	Baird *et al.* (2004)	0.2542	Baird *et al.* (2004)
Gd	*Hydrobia ulvae*	3HW	0.0602	Baird *et al.* (2004)	0.2542	Baird *et al.* (2004)
Bf	*Mytilus edulis*	0HW	0.0171	Baird *et al.* (2004)	0.8100	Fig. 5 in Widdows & Bayne (1971)
Bf	*Mytilus edulis*	1HW	0.0265	Baird *et al.* (2004)	0.8100	Fig. 5 in Widdows & Bayne (1971)
Bf	*Mytilus edulis*	3HW	0.0265	Baird *et al.* (2004)	0.8100	Fig. 5 in Widdows & Bayne (1971)
Bf	*Mya truncata*	0HW	0.0409	Baird *et al.* (2004); from *Mya arenaria*	0.6652	Baird *et al.* (2004); see *Mya arenaria*
Bf	*Mya truncata*	1HW	0.0648	Baird *et al.* (2004); from *Mya arenaria*	0.6652	Baird *et al.* (2004); see *Mya arenaria*
Bf	*Mya truncata*	3HW	0.0648	Baird *et al.* (2004); from *Mya arenaria*	0.6652	Baird *et al.* (2004); see *Mya arenaria*
Bf	*Macoma balthica*	0HW	0.0836	Baird *et al.* (2004)	0.1835	Baird *et al.* (2004)
Bf	*Macoma balthica*	1HW	0.1070	Baird *et al.* (2004)	0.1835	Baird *et al.* (2004)
Bf	*Macoma balthica*	3HW	0.1070	Baird *et al.* (2004)	0.1835	Baird *et al.* (2004)
Po	*Hediste diversicolor*	0HW	0.0038	Baird *et al.* (2004)	0.8080	Neuhoff (1979)
Po	*Hediste diversicolor*	1HW	0.0044	Baird *et al.* (2004)	0.8080	Neuhoff (1979)
Po	*Hediste diversicolor*	3HW	0.0044	Baird *et al.* (2004)	0.8080	Neuhoff (1979)
Pd	*Marenzelleria viridis*	0HW	0.0126	Zettler (1997)	0.7402	Baird *et al.* (2004); see *P. elegans*
Pd	*Marenzelleria viridis*	1HW	0.0220	Zettler (1997)	0.7402	Baird *et al.* (2004); see *P. elegans*
Pd	*Marenzelleria viridis*	3HW	0.0220	Zettler (1997)	0.7402	Baird *et al.* (2004); see *P. elegans*
Pd	*Pygospio elegans*	0HW	0.0079	Baird *et al.* (2004)	0.7402	Baird *et al.* (2004)
Pd	*Pygospio elegans*	1HW	0.0112	Baird *et al.* (2004)	0.7402	Baird *et al.* (2004)
Pd	*Pygospio elegans*	3HW	0.0112	Baird *et al.* (2004)	0.7402	Baird *et al.* (2004)
Pd	*Polydora cornuta*	0HW	0.0079	Baird *et al.* (2004); see *P. elegans*	0.7402	Baird *et al.* (2004); see *P. elegans*
Pd	*Polydora cornuta*	1HW	0.0112	Baird *et al.* (2004); see *P. elegans*	0.7402	Baird *et al.* (2004); see *P. elegans*
Pd	*Polydora cornuta*	3HW	0.0112	Baird *et al.* (2004); see *P. elegans*	0.7402	Baird *et al.* (2004); see *P. elegans*

Table S3. Respiration to biomass (R/B) ratios of consumers were either measured at the end of the final heat wave (i.e., *Idotea balthica*, *Gammarus salinus*, *Gammarus locusta* and *Littorina littorea*) or obtained from the literature. R/B ratios derived from the literature reflect the temperature of the treatments (i.e., 20 °C in case of 0HW, and 25 °C for 1HW and 3HW) because the values were corrected using Q10 parameters (Table S4). The R/B ratios (day^{-1}) served, together with the production to biomass (P/B) ratios and assimilation efficiencies (α; Table S2), to quantify the total consumption (Q) of the heterotrophic compartments. Average values per species are reported. When a value for a species was unavailable, the value from a similar taxon was used; the proxies employed are indicated in the reference column.

Code	Species	0HW	1HW	3HW	References
Io	*Idotea balthica*	0.1426	0.0674	0.1401	Ito *et al.* (2019)
Ao	*Gammarus salinus*	0.0761	0.1166	0.0407	Ito *et al.* (2024)
Ao	*Gammarus locusta*	0.0724	0.1025	0.1030	Ito *et al.* (2024)
Gh	*Littorina littorea*	0.0352	0.0794	0.0288	Ito *et al.* (2024)
Id	*Jaera albifrons*	0.1426	0.0674	0.1401	Ito *et al.* (2024); see *I. balthica*
Ad	*Corophium volutator*	0.0512	0.0794	0.0794	Baird *et al.* (2004); rescaled to treatment temperature with Q10
Ad	*Microdeutopus* sp.	0.0512	0.0794	0.0794	Baird *et al.* (2004); see *C. volutator*
Gd	*Hydrobia ulvae*	0.0705	0.0928	0.0928	Baird *et al.* (2004); rescaled to treatment temperature with Q10
Bf	*Mytilus edulis*	0.0943	0.1458	0.1458	Baird *et al.* (2004); rescaled to treatment temperature with Q10
Bf	*Mya truncata*	0.0952	0.1508	0.1508	Baird *et al.* (2004); from *Mya arenaria*
Bf	*Macoma balthica*	0.0156	0.0199	0.0199	Baird *et al.* (2004); rescaled to treatment temperature with Q10
Po	*Hediste diversicolor*	0.0155	0.0176	0.0176	Baird *et al.* (2004); rescaled to treatment temperature with Q10
Pd	*Marenzelleria viridis*	0.0366	0.0517	0.0517	Baird *et al.* (2004); see *P. elegans*
Pd	*Pygospio elegans*	0.0366	0.0517	0.0517	Baird *et al.* (2004); rescaled to treatment temperature with Q10
Pd	*Polydora cornuta*	0.0366	0.0517	0.0517	Baird *et al.* (2004); see *P. elegans*

Table S4. Q10 values applied to scale the metabolic parameters that were not quantified from direct measurements during the mesocosm experiment. The use of Q10 values allowed obtaining R/B and P/B ratios expressed for the temperature corresponding to the three treatments (i.e., 20 °C in case of 0HW, and 25 °C for 1HW and 3HW). The R/B and P/B ratios were corrected using the equation: $ratio_{HW} = ratio_{lit} \cdot Q10^{(T_{HW}-T_{lit})/10\ °C}$. Several Q10 values were taken from de la Vega *et al.* (2018) and references cited therein, as indicated below. When a value for a species was unavailable, the value from a similar taxon was used; the proxies employed are indicated in the reference column.

**Code**	**Species**	**Q10**	**References**
Io	*Idotea balthica*	4.02	Salomon & Buchholz (2000)
Ao	*Gammarus salinus*	2.40	Halcrow & Boyd (1967)
Ao	*Gammarus locusta*	2.40	Halcrow & Boyd (1967)
Gh	*Littorina littorea*	1.15	Newell & Pye (1971)
Id	*Jaera albifrons*	4.02	See *I. balthica*
Ad	*Corophium volutator*	2.40	See *Gammarus* sp.
Ad	*Microdeutopus* sp.	2.40	See *Gammarus* sp.
Gd	*Hydrobia ulvae*	1.73	Asmus (1984)
Bf	*Mytilus edulis*	2.39	Head (1962); Widdows (1973)
Bf	*Mya truncata*	2.51	Value of *Mya arenaria*; Kennedy & Mihursky (1972)
Bf	*Macoma balthica*	1.64	Asmus (1984)
Po	*Hediste diversicolor*	1.29	Kristensen (1983)
Pd	*Marenzelleria viridis*	3.05	Lehtonen (2004)
Pd	*Pygospio elegans*	2.00	Timmermann *et al.* (2012)
Pd	*Polydora cornuta*	2.00	Same as *P. elegans*

Table S5. Information on the resource and respective consumers that was used for depicting the trophic interactions that compose the ecological network of this study. The information was retrieved from literature (Reference column).

Resource	Consumer	Reference
POC	Ad	*Microdeutopus gryllotalpa*: Köhn & Gosselck, 1989; DeWitt & Levinton, 1985
ZM	Ao	*Gammarus* sp.: Andersson et al. 2009; Spooner 1947
FV	Ao	*Gammarus* sp.: Andersson et al. 2009; Spooner 1947
FA	Ao	*Gammarus* sp.: Andersson et al. 2009; Spooner 1947
POC	Ao	*Gammarus* sp.: Andersson et al. 2009; Spooner 1947
POC	Id	*Jaera albifrons*: Jones 1972
ZM	Io	*Idotea balthica*: Leidenberger et al. 2012
FV	Io	*Idotea balthica*: Leidenberger et al. 2012
FA	Io	*Idotea balthica*: Leidenberger et al. 2012
POC	Io	*Idotea balthica*: Leidenberger et al. 2012
ZM	Gh	*Littorina littorea*: Jagnow and Gosselck 1987
FV	Gh	*Littorina littorea*: Jagnow and Gosselck 1987
FA	Gh	*Littorina littorea*: Jagnow and Gosselck 1987
POC	Gh	*Littorina littorea*: Jagnow and Gosselck 1987
POC	Bf	*Mytilus edulis, Mya* sp.: Jagnow and Gosselck 1987; Winter 1969
POC	Pd	*Marenzelleria viridis; Polydora cornuta; Pygospio elegans*: Kotta and Ólafsson 2003; Takata et al. 2011; Brey 1991
Ad	Po	*Hediste diversicolor*: Bick and Gosselck 1985
Ao	Po	*Hediste diversicolor*: Bick and Gosselck 1985
Gh	Po	*Hediste diversicolor*: Bick and Gosselck 1985
Bf	Po	*Hediste diversicolor*: Bick and Gosselck 1985
Pd	Po	*Hediste diversicolor*: Bick and Gosselck 1985
Po	Po	*Hediste diversicolor*: Bick and Gosselck 1985
Gd	Po	*Hediste diversicolor*: Bick and Gosselck 1985

POC	Po	*Hediste diversicolor*: Bick and Gosselck 1985
POC	Gd	*Hydrobia* sp.: Aberle et al. 2009; Jagnow and Gosselck 1987

Table S6. Summary of network-derived indices. The symbol T_{ij} indicates the carbon flowing from compartment *i* to compartment *j* while (1) $T_{i\cdot}$ is the total carbon leaving from compartment *i* and (2) $T_{\cdot j}$ represents the total carbon entering the compartment *j*. The ecosystem is composed of *n* compartments and the matrix with all flows, including those reporting exchanges with the external environment, has size *n*+3. In the matrix, (1) the index 0 = imports, (2) indices from 1 to *n* = ecosystem compartments, (3) the index *n*+1 = respiration, and (4) the index *n*+2 refers to the exports (see Figure S1 in Scotti *et al.*, 2009). In the equations, *k* is a scalar constant while TST_{flow} is the sum of all internal flows, excluding those crossing the ecosystem boundaries ($TST_{flow} = \sum_{i=1}^{n}\sum_{j=1}^{n} T_{ij}$). Indices refer to acronym and the full name of each index, Equation details the calculations applied in this study, Ref. are the references of the equations, and Definition and Relevance summarize ecological meaning and importance of each index. Response to heat waves condense the findings for each index; thus, upward arrows (⇑) indicate the increase and the downward arrows (⇓) the decrease of the index of the systems exposed to the heat wave treatments (1HW and 3HW) in relation to 0HW treatment. Significance of the trends was highlighted with either asterisks (*) ($p < 0.05$) or solid circles (●) ($0.05 \leq p < 0.10$).

**Indices**		**Equation**	**Definition and relevance**	**Refs.**	**Response to heat waves**	
					**1HW**	**3HW**
TST	total system throughput	$TST = \sum_{i=0}^{n+2}\sum_{j=0}^{n+2} T_{ij}$	Total amount of carbon flowing through a system. The fluxes included are imports, intercompartmental exchanges, respiration, and exports.<br>Changes in TST indicate how ecosystem activity (i.e., size) responds to disturbance.	Ulanowicz (1986)	⇑	⇓
H	flow diversity	$H = AMI + Hc$<br>or<br>$H = -k\sum_{i=0}^{n+2}\sum_{j=0}^{n+2}\left(\frac{T_{ij}}{\mathrm{TST}}\right)\cdot\log\left(\frac{T_{ij}}{TST}\right)$	Flow diversity indicates the evenness of the flux distribution in the ecosystem.<br>It depends on both the number of interactions and the relative carbon strength distribution.	Ulanowicz (2004)	⇓	⇑

AMI	average mutual information	$AMI = A \div TST$<br>or<br>$AMI = k\sum_{i=0}^{n+2}\sum_{j=0}^{n+2}\left(\frac{T_{ij}}{TST}\right)\cdot\log\left(\frac{T_{ij}\cdot TST}{T_{i.}\cdot T_{.j}}\right)$	AMI represents the efficiency through which carbon circulates in the ecosystem.<br>It quantifies the degrees of network organization and represents carbon transfer efficiency in the ecosystem.	Scotti *et al.* (2009)	⇑	⇑
Hc	residual diversity	$Hc = O \div TST$<br>or<br>$Hc = -k\sum_{i=0}^{n+2}\sum_{j=0}^{n+2}\left(\frac{T_{ij}}{TST}\right)\log\left(\frac{T_{ij}^2}{T_{i.}T_{.j}}\right)$	Hc informs about the redundancy of the routes pertaining carbon circulation in the network.<br>It represents the degrees of freedom preserved by the ecosystem and corresponds to stability; redundant pathways confer a higher capability to withstand disturbances.	Scotti *et al.* (2009)	⇓	⇓
DC	development capacity	$DC = TST \cdot H$<br>or<br>$DC = -\sum_{i=0}^{n+2}\sum_{j=0}^{n+2}T_{ij}\cdot\log\left(\frac{T_{ij}}{TST}\right)$<br>or<br>$DC = A + O$	DC reveals the upper threshold of development an ecosystem can attain.<br>It derives from ecosystem activity (i.e., size; TST) and a structural component informing on flow diversity (H).	Ulanowicz (2004)	⇑	⇓
A	ascendency	$A = TST \cdot AMI$<br>or<br>$A = \sum_{i=0}^{n+2}\sum_{j=0}^{n+2}T_{ij}\cdot\log\left(\frac{T_{ij}\cdot TST}{T_{i.}\cdot T_{.j}}\right)$	Ascendency is obtained by multiplying the ecosystem activity (i.e., size; TST) by the metric informing on the degree of carbon flow organization in the structure of links that connect the compartments (AMI).	Ulanowicz (2004)	⇑•	⇑

			It describes how efficiently the system processes the carbon flowing in it.			
O	overhead	$O = TST \cdot Hc$<br>or<br>$O = -\sum_{i=0}^{n+2}\sum_{j=0}^{n+2} T_{ij} \cdot \log\left(\frac{T_{ij}^2}{T_{i.}T_{.j}}\right)$<br>or<br>$O = OI + OE + OD + R$	It is calculated as the product of the system's degrees of freedom for carbon circulation (Hc) and its activity (i.e., size; TST).<br>O indicates the extent of pathway multiplicity in the whole network.	Ulanowicz (2004)	⇑	⇓
IHc	internal residual diversity	$IHc = R \div TST$	IHc informs about the redundancy in the routes responsible for carbon fluxes in the system (structural component only).	Scotti *et al.* (2022)	⇓	⇑
OI	overhead on imports	$OI = -\sum_{j=1}^{n} T_{0,j} \cdot \log\left(\frac{T_{0,j}^2}{T_{0.}\,T_{.j}}\right)$	OI indicates the degrees of redundancy in carbon imports. Higher OI values correspond to more evenly distributed GPP.	Ulanowicz (2004)	⇑	⇓
OE	overhead on exports	$OE = -\sum_{i=1}^{n} T_{i,n+2} \cdot \log\left(\frac{T_{i,n+2}^2}{T_{i.}\,T_{.n+2}}\right)$	OE indicates the degrees of redundancy in carbon exports. Higher OE values correspond to more evenly distributed exports.	Ulanowicz (2004)	⇑	⇓
OD	dissipation overhead	$OD = -\sum_{i=1}^{n} T_{i,n+1} \cdot \log\left(\frac{T_{i,n+1}^2}{T_{i.}\,T_{.n+1}}\right)$	OD indicates the degrees of evenness in the contribution of compartments to the total respiration. Higher OD values correspond to more evenly distributed respiration flows.	Ulanowicz (2004)	⇑	⇑

R	redundancy	$R = -\sum_{i=1}^{n}\sum_{j=1}^{n} T_{ij} \cdot \log\left(\frac{T_{ij}^2}{T_{i.}\,T_{.j}}\right)$	R indicates the degrees of uncertainty in carbon circulation within the ecosystem. Higher values correspond to more evenly distributed carbon flows within the system.	Ulanowicz (2004); Safi *et al.* (2019)	⇑*	⇑●
relA	relative ascendency	$relA = A \div DC$	The relative ascendency quantifies the fraction of organized complexity within an ecosystem. It ranges between 0 and 1.<br>Higher values indicate lower degrees of freedom in carbon circulation. Organization is related to a lower number of redundant pathways, making the system more efficient in transferring carbon. Efficiency comes at the expense of stability and resilience.	Scotti *et al.* (2022)	⇑	⇑
relO	relative overhead	$relO = O \div DC$	The relative overhead informs on the proportion of redundant flows within an ecosystem. It ranges between 0 and 1.<br>It expresses the resilience of the system. Multiple, redundant pathways increase the chances to recover from disturbances. For instance, trophic groups may shift their diets among alternative prey items of equivalent importance in case of perturbations.	Scotti *et al.* (2022)	⇓	⇓
IA	internal ascendency	$IA = \sum_{i=1}^{n}\sum_{j=1}^{n} T_{ij} \cdot \log\left(\frac{T_{ij} \cdot TST_{flow}}{T_{i.} \cdot T_{.j}}\right)$	IA is the product of total internal activity (TST_{flow}) and the internal level of flow organization (AMI calculated using the flows among compartments only).	Ulanowicz (2004)	⇑	⇓

			It quantifies how efficiently the ecosystem processes circulating carbon.			
IR	Internal relative redundancy	$IR = R \div IDC$	Proportion of internal redundancy. It is the proportion of carbon transferred through redundant pathways within the ecosystem (it is a proxy for resilience).	Scotti *et al.* (2022)	⇓	⇑
IDC	internal development capacity	$IDC = -\sum_{i=1}^{n}\sum_{j=1}^{n} T_{ij} \cdot \log\left(\frac{T_{ij}}{TST_{flow}}\right)$	IDC reveals the upper threshold of development the ecosystem can attain, without considering the exchanges with the external environment (i.e., excluding imports, exports, and respiration).	Scotti *et al.* (2022)	⇑*	⇑•
ICC	intercompartmental connectance	$ICC = e^{\left(\frac{-k\sum_{i=1}^{n}\sum_{j=1}^{n}\frac{T_{ij}}{TST_{flow}}\cdot\log\left(\frac{T_{ij}^2}{T_{i.}T_{.j}}\right)}{2}\right)}$	It represents a link-weighted measure of the complexity of interactions in the ecosystem.<br>The expectation is that disturbance results in lower connectance.	Ulanowicz & Wolff (1991)	⇓	⇓•
FWC	food web connectance	$FWC = e^{\left(\frac{-k\sum_{i=1}^{n-1}\sum_{j=1}^{n-1}\frac{T_{ij}}{TST_{flow}}\cdot\log\left(\frac{T_{ij}^2}{T_{i.}T_{.j}}\right)}{2}\right)}$	It indicates the connectivity between the living compartments only. Therefore, it is a link-weighted connectance considering food web compartments and excluding the POC.	Ulanowicz & Wolff (1991)	⇓	⇓
ART	average residence time	$ART = \frac{\sum biomass}{\sum respiration + \sum export}$	Average time that one unit of carbon takes to circulate before leaving the ecosystem. ART is related to the ability of the system to retain carbon. It is analogous to the turnover time in biogeochemical models and systems theory.	Christensen (1995)	⇓•	⇓

Table S7. Ecosystem attributes. Details on their calculation are provided together with information on the responses to experimental heat waves. Response to heat waves condense the findings for each attribute; thus, upward arrows (⇑) indicate the increase and the downward arrows (⇓) the decrease of the attribute of the systems exposed to the heat wave treatments (1HW and 3HW) in relation to 0HW treatment. Significance of the trends was highlighted with either asterisks (*) ($p < 0.05$) or solid circles (●) ($0.05 \leq p < 0.10$).

Attributes		Definition and relevance	Response to heat waves	
			1HW	3HW
GPP	gross primary production	It represents the sum of the gross primary production of all macrophytes.	⇑	⇓
Resp	respiration	Sum of the respiration (i.e., loss of carbon) from all compartments.	⇑●	⇑
Export		Total usable carbon that is transported beyond the boundaries of the ecosystem.	⇓	⇓
MB	macrophytes' biomass	Total biomass of macrophytes, i.e. seagrasses and macroalgae.	⇑	⇓
CB	consumers' biomass	Total biomass of consumers.	⇑	⇑●
Detritivory		The sum of carbon flowing from detritus to consumers.	⇑*	⇑*
Herbivory		The sum of carbon flowing from macrophytes to consumers.	⇑	⇑
DH	detritivory:herbivory ratio	Ratio between detritivory and herbivory.	⇑●	⇑

POC	particulate organic carbon	Total POC biomass.	⇓	⇑
POCsink	flow to particulate organic carbon	The sum of carbon flowing from all compartments to detritus (i.e., egestion of heterotrophs).	⇑*	⇑

References

Aberle, N., Malzahn, A.M., Grey, J., Hillebrand, H. and Wiltshire, K.H. 2009. Altered complementary feeding strategies of the consumers *Hydrobia ulvae* and *Idotea emarginata* via passive selectivity. *Helgoland Marine Research,* 63: 189–197. https://doi.org/10.1007/s10152-009-0148-9

Andersson, S., Persson, M., Moksnes, P.-O. and Baden, S. 2009. The role of the amphipod Gammarus locusta as a grazer on macroalgae in Swedish seagrass meadows. *Marine Biology*, 156: 969–981. https://doi.org/10.1007/s00227-009-1141-1

Asmus, H., 1984. Freilanduntersuchungen zur Sekundärproduktion und Respiration benthischer Gemeinschaften im Wattenmeer der Nordsee (No. 122). *Institut für Meereskunde an der Christian-Albrecht-Universität Kie*l.

Baden, S.P. and Pihl, L., 1984. Abundance, biomass and production of mobile epibenthic fauna in *Zostera marina* (L.) meadows, western Sweden. *Ophelia* 23, 65-90. https://doi.org/10.1080/00785236.1984.10426605

Baird, D., Asmus, H. and Asmus, R., 2004. Energy flow of a boreal intertidal ecosystem, the Sylt-Rømø Bight. *Marine Ecology Progress Series*, 279, 45-61. https://doi.org/10.3354/meps279045

Bick, A. and Gosselck, F. 1985. Arbeitsschlüssel zur Bestimmung der Polychaeten der Ostsee. *Zoosystematics & Evolution* 61: 171–272. https://doi.org/10.1002/mmnz.19850610202

Brey, T. 1991. Interactions in soft bottom benthic, communities: quantitative aspects of behaviour in the surface deposit feeders *Pygospio elegans* (Polychaeta) and *Macoma balthica* (Bivalvia). *Helgoländer Meeresuntersuchungen*, 45, 301–316 (1991). https://doi.org/10.1007/BF02365522

Christensen, V., 1995. Ecosystem maturity—towards quantification. *Ecological Modelling*, 77(1), 3-32. https://doi.org/10.1016/0304-3800(93)E0073-C

de la Vega, C., Horn, S., Baird, D., Hines, D., Borrett, S., Jensen, L.F., Schwemmer, P., Asmus, R., Siebert, U. and Asmus, H., 2018. Seasonal dynamics and functioning of the Sylt-Rømø Bight, northern Wadden Sea. *Estuarine, Coastal and Shelf Science*, 203, pp.100-118. https://doi.org/10.1016/j.ecss.2018.01.021

DeWitt, T.H. and Levinton, J.S. 1985. Disturbance, emigration, and refugia: How the mud snail, *Ilyanassa obsoleta* (Say), affects the habitat distribution of an epifaunal amphipod, *Microdeutopus*

gryllotalpa (Costa). *Journal of Experimental Marine Biology and Ecology*, 92(1), pp.97-113. https://doi.org/10.1016/0022-0981(85)90024-3

Grahame, J., 1973. Assimilation Efficiency of *Littorina littorea* (L.) (Gastropoda: Prosobranchiata). *The Journal of Animal Ecology* 42, 383. https://doi.org/10.2307/3292

Halcrow, K., Boyd, C.M., 1967. The oxygen consumption and swimming activity of the amphipod *Gammarus oceanicus* at different temperatures. *Comparative Biochemistry and Physiology* 23, 233-242. https://doi.org/10.1016/0010-406X(67)90491-4

Hansen, T., Burmeister, A. and Sommer, U., 2009. Simultaneous $\delta^{13}C$, $\delta^{15}N$ and $\delta^{34}S$ measurements of low-biomass samples using a technically advanced high sensitivity elemental analyzer connected to an isotope ratio mass spectrometer. *Rapid Commun. Mass Spectrom*, *23*, pp.3387-3393. https://doi.org/10.1002/rcm.4267

Head, K.R., 1962. Respiration of the bivalved molluscs *Mytilus edulis* L. and *Brachidontes demissus plicatulus* Lamarck as a function of size and temperature. *Comparative Biochemistry and Physiology*, *7*(1-2), pp.89-101. https://doi.org/10.1016/0010-406X(62)90031-2

Ito, M., Guy-Haim, T., Sawall, Y., Franz, M., Buchholz, B., Hansen, T., Neitzel, P., Pansch, C., Steinhoff, T., Wahl, M. and Weinberger, F., 2024. Responses at various levels of ecological hierarchy indicate acclimation to sequential sublethal heatwaves in a temperate benthic ecosystem. *Philosophical Transactions B*, 379(1909), p.20230171. https://doi.org/10.1098/rstb.2023.0171

Ito, M., Halouani, G., Cresson, P., Giraldo, C. and Girardin, R., 2023. Detection of fishing pressure using ecological network indicators derived from ecosystem models. *Ecological Indicators*, *147*, p.110011. https://doi.org/10.1016/j.ecolind.2023.110011

Ito, M., Scotti, M., Franz, M., Barboza, F.R., Buchholz, B., Zimmer, M., Guy-Haim, T. and Wahl, M., 2019. Effects of temperature on carbon circulation in macroalgal food webs are mediated by herbivores. *Marine Biology*, 166(12), p.158. https://doi.org/10.1007/s00227-019-3596-z

Jagnow, B. and Gosselck, F., 1987. Bestimmungsschlüssel für die Gehäuseschnecken und Muscheln der Ostsee. *Mitteilungen aus dem Museum für Naturkunde in Berlin. Zoologisches Museum und Institut für Spezielle Zoologie (Berlin),* 63(2), pp.191-268. https://doi.org/10.1002/mmnz.19870630202

Jones, M.B., 1972. The mouthparts of the members of the Jaera albifrons group of species (Crustacea: Isopoda). *Marine Biology*, 14(3), pp.264-270. https://doi.org/10.1007/BF00348290

Kennedy, V.S. and Mihursky, J.A., 1972. Effects of temperature on the respiratory metabolism of three Chesapeake Bay bivalves. *Chesapeake Science*, *13*(1), pp.1-22. https://doi.org/10.2307/1350546

Köhn, J. and Gosselck, F., 1989. Bestimmungsschlüssel der Malakostraken der Ostsee. *Mitteilungen aus dem Museum für Naturkunde in Berlin. Zoologisches Museum und Institut für Spezielle Zoologie (Berlin)*, 65(1), pp.3-114. https://doi.org/10.1002/mmnz.19890650102

Kotta, J. and Ólafsson, E., 2003. Competition for food between the introduced polychaete *Marenzelleria viridis* (Verrill) and the native amphipod *Monoporeia affinis* Lindström in the Baltic Sea. *Journal of Sea Research*, 50(1), pp.27-35. https://doi.org/10.1016/S1385-1101(03)00041-8

Kraemer, G.P. and Alberte, R.S., 1993. Age-related patterns of metabolism and biomass in subterranean tissues of *Zostera marina* (eelgrass). *Marine Ecology Progress Series*, *95*, pp.193-203. https://doi.org/10.3354/meps095193

Kristensen, E., 1983. Ventilation and oxygen uptake by three species of *Nereis* (Annelida: Polychaeta). II. Effects of temperature and salinity changes. *Marine Ecology Progress Series*, *12*, pp.299-306. https://doi.org/10.3354/meps012299

Küppers, U. and Kremer, B.P., 1978. Longitudinal profiles of carbon dioxide fixation capacities in marine macroalgae. *Plant Physiology*, *62*(1), pp.49-53. https://doi.org/10.1104/pp.62.1.49

Lehtonen, K.K., 2004. Seasonal variations in the physiological condition of the benthic amphipods *Monoporeia affinis* and *Pontoporeia femorata* in the Gulf of Riga (Baltic Sea). *Aquatic Ecology* 38(3), pp.441-456. https://doi.org/10.1023/B:AECO.0000035165.97619.78

Leidenberger, S., Harding, K. and Jonsson, P.R., 2012. Ecology and distribution of the isopod genus *Idotea* in the Baltic Sea: key species in a changing environment. *Journal of crustacean biology*, 32(3), pp.359-389. https://doi.org/10.1163/193724012X626485

Neuhoff, H.G., 1979. Influence of temperature and salinity on food conversion and growth of different *Nereis* species (Polychaeta, Annelida). *Marine Ecology Progress Series*, 1, pp.255-262. https://doi.org/10.3354/meps001255

Newell, R.C. and Pye, V.I., 1971. Quantitative aspects of the relationship between metabolism and temperature in the winkle *Littorina littorea* (L.). *Comparative Biochemistry and Physiology Part B: Comparative Biochemistry*, *38*(4), pp.635-650. https://doi.org/10.1016/0305-0491(71)90267-7

Nielsen, M.V. and Kofoed, L.H., 1982. Selective feeding and epipsammic browsing by the deposit-feeding amphipod *Corophium volutator*. *Marine Ecology Progress Series*, *10*, pp.81-88. https://doi.org/10.3354/meps010081

Redant, F., 1898. Productivity of epibenthic species: A review (ICES CM Document No. ICES CM 1989/L:2), Biological Oceanography Committee (Ref.: Shellfish Committee). International Council for the Exploration of the Sea (ICES), Copenhagen.

Rumohr, H., Brey, T. and Ankar, S., 1987. A compilation of biometric conversion factors for benthic invertebrates of the Baltic Sea. *Institut für Meereskunde*.

Safi, G., Giebels, D., Arroyo, N.L., Heymans, J.J., Preciado, I., Raoux, A., Schückel, U., Tecchio, S., de Jonge, V.N. and Niquil, N., 2019. Vitamine ENA: a framework for the development of ecosystem-based indicators for decision makers. *Ocean & Coastal Management*, 174, pp.116-130. https://doi.org/10.1016/j.ocecoaman.2019.03.005

Salomon, M. and Buchholz, F., 2000. Effects of temperature on the respiration rates and the kinetics of citrate synthase in two species of *Idotea* (Isopoda, Crustacea). *Comparative Biochemistry and Physiology Part B: Biochemistry and Molecular Biology*, *125*(1), pp.71-81. https://doi.org/10.1016/S0305-0491(99)00158-3

Scotti, M., Bondavalli, C., Bodini, A. and Allesina, S., 2009. Using trophic hierarchy to understand food web structure. *Oikos*, 118(11), pp.1695-1702. https://doi.org/10.1111/j.1600-0706.2009.17073.x

Scotti, M., Bondavalli, C., Rossetti, G. and Bodini, A., 2022. Flow network indices signal a directional change in ecosystems: Evidence from a small mountain lake (Lake Santo, northern Italy). *Ecological Indicators*, *139*, p.108896. https://doi.org/10.1016/j.ecolind.2022.108896

Spooner, G.M., 1947. The distribution of Gammarus species in estuaries. Part I. Journal of the Marine Biological Association of the United Kingdom, 27(1), pp.1-52. https://doi.org/10.1017/S0025315400014107

Stock, B.C., Jackson, A.L., Ward, E.J., Parnell, A.C., Phillips, D.L. and Semmens, B.X., 2018. Analyzing mixing systems using a new generation of Bayesian tracer mixing models. *PeerJ*, *6*, p.e5096. https://doi.org/10.7717/peerj.5096

Takata, N., Takahashi, H., Ukita, S., Yamasaki, K. and Awakihara, H., 2011. Ecology of P*olydora cornuta* Bosc, 1802 (Spionidae: Polychaeta) in the eutrophic port of Fukuyama, with special

reference to life cycle, distribution, and feeding type. *Journal of Water and Environment Technology*, 9(3), pp.259-275. https://doi.org/10.2965/jwet.2011.259

Timmermann, K., Norkko, J., Janas, U., Norkko, A., Gustafsson, B.G. and Bonsdorff, E., 2012. Modelling macrofaunal biomass in relation to hypoxia and nutrient loading. *Journal of Marine Systems*, *105*, pp.60-69. https://doi.org/10.1016/j.jmarsys.2012.06.001

Ulanowicz, R.E., 1986. Growth and Development: Ecosystems Phenomenology. Springer-Verlag, New York, 203 pp.

Ulanowicz, R.E., 2004. Quantitative methods for ecological network analysis. *Computational biology and chemistry*, *28*(5-6), pp.321-339. https://doi.org/10.1016/j.compbiolchem.2004.09.001

Ulanowicz, R.E. and Wolff, W.F., 1991. Ecosystem flow networks: loaded dice? *Mathematical Biosciences*, 103(1), pp.45-68. https://doi.org/10.1016/0025-5564(91)90090-6

Widdows, J., 1973. The effects of temperature on the metabolism and activity of *Mytilus edulis*. *Netherlands Journal of Sea Research*, 7, pp.387-398. https://doi.org/10.1016/0077-7579(73)90060-4

Widdows, J. and Bayne, B.L., 1971. Temperature acclimation of Mytilus edulis with reference to its energy budget. *Journal of the Marine Biological Association of the United Kingdom*, *51*(4), pp.827-843. https://doi.org/10.1017/S0025315400018002

Winter, J.E., 1969. Über den Einfluß der Nahrungskonzentration und anderer Faktoren auf Filtrierleistung und Nahrungsausnutzung der Muscheln *Arctica islandica* und *Modiolus modiolus*. *Marine Biology*, 4(2), pp.87-135. https://doi.org/10.1007/BF00347037

Zettler, M.L., 1997. Population dynamics, growth and production of the neozoon *Marenzelleria* cf. *viridis* (Verrill, 1873)(Polychaeta: Spionidae) in a coastal water of the southern Baltic Sea. *Aquatic Ecology*, *31*(2), pp.177-186. https://doi.org/10.1023/A:1009903521182
