# Supplementary material for: Network indices show heat wave‐induced fragility in experimental food webs: Operational insights for ecosystem management
Source: Ecol Appl. 2026 Sep 8;36(6):e70277. doi: 10.1002/eap.70277 (PMC13554289; doi:10.1002/eap.70277)
Supplement: Supplementary file 2 [file EAP-36-e70277-s002.pdf]

Network indices show heat wave-induced fragility in experimental food webs: Operational insights for ecosystem management

Maysa Ito, Anna Waffender, and Marco Scotti

Ecological Applications

Appendix S2: Results

Table S1. Results of the GLM fitted to the ENA-derived indices. Significant increases of the treatment (1HW or 3HW) in relation to the 0HW are represented as green cells and marginally significant changes are highlighted in yellow cells.

<table>
<tr><th>Response variable</th><th>Predictors</th><th>Estimates</th><th>Std. Error</th><th>t-value</th><th>Pr(>|t|)</th></tr>
<tr><td rowspan="5">TST</td><td>(Intercept)</td><td>2.43E-04</td><td>4.65E-05</td><td>5.228</td><td>0.000794</td></tr>
<tr><td>1HW</td><td>-7.19E-05</td><td>5.69E-05</td><td>-1.263</td><td>0.242147</td></tr>
<tr><td>3HW</td><td>1.64E-05</td><td>7.38E-05</td><td>0.222</td><td>0.829841</td></tr>
<tr><td colspan="2">Null deviance:</td><td>1.5721</td><td>df</td><td>10</td></tr>
<tr><td colspan="2">Residual deviance:</td><td>1.1911</td><td>df</td><td>8</td></tr>
<tr><td rowspan="5">H</td><td>(Intercept)</td><td>3.23E-01</td><td>1.37E-02</td><td>23.644</td><td>1.09E-08</td></tr>
<tr><td>1HW</td><td>1.59E-03</td><td>1.94E-02</td><td>0.082</td><td>0.937</td></tr>
<tr><td>3HW</td><td>-9.33E-03</td><td>2.05E-02</td><td>-0.454</td><td>0.662</td></tr>
<tr><td colspan="2">Null deviance:</td><td>0.059924</td><td>df</td><td>10</td></tr>
<tr><td colspan="2">Residual deviance:</td><td>0.057665</td><td>df</td><td>8</td></tr>
<tr><td rowspan="5">AMI</td><td>(Intercept)</td><td>8.10E-01</td><td>4.20E-02</td><td>19.296</td><td>5.40E-08</td></tr>
<tr><td>1HW</td><td>-1.86E-02</td><td>5.87E-02</td><td>-0.316</td><td>0.76</td></tr>
<tr><td>3HW</td><td>-6.74E-02</td><td>6.12E-02</td><td>-1.102</td><td>0.303</td></tr>
<tr><td colspan="2">Null deviance:</td><td>0.099911</td><td>df</td><td>10</td></tr>
<tr><td colspan="2">Residual deviance:</td><td>0.086369</td><td>df</td><td>8</td></tr>
<tr><td rowspan="5">Hc</td><td>(Intercept)</td><td>5.38E-01</td><td>3.29E-02</td><td>16.318</td><td>2.00E-07</td></tr>
<tr><td>1HW</td><td>1.31E-02</td><td>4.72E-02</td><td>0.277</td><td>0.789</td></tr>
<tr><td>3HW</td><td>5.82E-03</td><td>5.06E-02</td><td>0.115</td><td>0.911</td></tr>
<tr><td colspan="2">Null deviance:</td><td>0.12823</td><td>df</td><td>10</td></tr>
<tr><td colspan="2">Residual deviance:</td><td>0.12708</td><td>df</td><td>8</td></tr>
<tr><td rowspan="5">DC</td><td>(Intercept)</td><td>8.10E-05</td><td>1.40E-05</td><td>5.779</td><td>0.000415</td></tr>
<tr><td>1HW</td><td>-2.45E-05</td><td>1.71E-05</td><td>-1.432</td><td>0.189955</td></tr>
<tr><td>3HW</td><td>3.93E-08</td><td>2.14E-05</td><td>0.002</td><td>0.99858</td></tr>
<tr><td colspan="2">Null deviance:</td><td>1.27085</td><td>df</td><td>10</td></tr>
<tr><td colspan="2">Residual deviance:</td><td>0.93159</td><td>df</td><td>8</td></tr>
</table>

**A**	(Intercept)	2.08E-04	3.06E-05	6.788	0.000139
	1HW	-7.08E-05	3.67E-05	-1.932	0.089493
	3HW	-1.23E-05	4.52E-05	-0.271	0.793324
	Null deviance:		1.10002	df	10
	Residual deviance:		0.69432	df	8
**O**	(Intercept)	1.33E-04	2.58E-05	5.132	0.000894
	1HW	-3.65E-05	3.19E-05	-1.143	0.286055
	3HW	5.64E-06	4.04E-05	0.139	0.89264
	Null deviance:		1.4842	df	10
	Residual deviance:		1.1802	df	8
**IHc**	(Intercept)	2.91E+00	5.91E-01	4.922	0.00116
	1HW	4.76E-01	9.07E-01	0.525	0.61381
	3HW	-4.34E-01	8.29E-01	-0.523	0.61503
	Null deviance:		1.5237	df	10
	Residual deviance:		1.3539	df	8
**OI**	(Intercept)	8.21E+00	2.86E-01	28.751	2.32E-09
	1HW	2.83E-01	4.04E-01	0.7	0.504
	3HW	-2.62E-01	4.36E-01	-0.601	0.565
	Null deviance:		3.2976	df	10
	Residual deviance:		2.7895	df	8
**OE**	(Intercept)	5.70E+00	3.16E-01	18.044	9.13E-08
	1HW	2.33E-01	4.47E-01	0.521	0.616
	3HW	-5.20E-01	4.82E-01	-1.079	0.312
	Null deviance:		3.7993	df	10
	Residual deviance:		2.8793	df	8
**OD**	(Intercept)	4.15E-04	6.49E-05	6.395	0.00021
	1HW	-1.31E-04	7.86E-05	-1.664	0.13462
	3HW	-4.62E-05	9.30E-05	-0.497	0.63233
	Null deviance:		1.10004	df	10
	Residual deviance:		0.79746	df	8
**R**	(Intercept)	8.67E-04	8.04E-05	10.79	4.80E-06
	1HW	-2.52E-04	9.86E-05	-2.552	0.034
	3HW	-2.06E-04	1.07E-04	-1.926	0.0902
	Null deviance:		0.5212	df	10
	Residual deviance:		0.27134	df	8
**relA**	(Intercept)	2.51E+00	1.21E-01	20.775	3.02E-08
	1HW	-9.19E-02	1.68E-01	-0.547	0.599
	3HW	-1.52E-01	1.79E-01	-0.849	0.42
	Null deviance:		0.078623	df	10
	Residual deviance:		0.071712	df	8
**relO**	(Intercept)	1.66E+00	5.64E-02	29.442	1.92E-09
	1HW	4.27E-02	8.08E-02	0.528	0.612
	3HW	7.35E-02	8.84E-02	0.832	0.43
	Null deviance:		0.04147	df	10
	Residual deviance:		0.038134	df	8

IA	(Intercept)	2.02E+00	6.71E-02	30.115	1.60E-09
	1HW	-8.47E-02	9.29E-02	-0.911	0.389
	3HW	5.68E-02	1.04E-01	0.546	0.6
		Null deviance:	0.045394	df	10
		Residual deviance:	0.036451	df	8
IR	(Intercept)	1.98E+00	6.49E-02	30.493	1.45E-09
	1HW	8.86E-02	9.39E-02	0.944	0.373
	3HW	-5.17E-02	9.77E-02	-0.529	0.611
		Null deviance:	0.042666	df	10
		Residual deviance:	0.033747	df	8
IDC	(Intercept)	4.40E-04	3.62E-05	12.15	1.95E-06
	1HW	-1.43E-04	4.37E-05	-3.275	0.0113
	3HW	-9.66E-05	4.88E-05	-1.98	0.083
		Null deviance:	0.52578	df	10
		Residual deviance:	0.21621	df	8
ICC	(Intercept)	3.79E-01	9.91E-03	38.281	2.38E-10
	1HW	2.76E-02	1.45E-02	1.898	0.0942
	3HW	9.63E-03	1.54E-02	0.626	0.5484
		Null deviance:	0.03174	df	10
		Residual deviance:	0.021692	df	8
FWC	(Intercept)	3.93E-01	1.27E-02	30.84	1.33E-09
	1HW	3.42E-02	1.88E-02	1.821	0.106
	3HW	1.23E-03	1.95E-02	0.063	0.951
		Null deviance:	0.051966	df	10
		Residual deviance:	0.034856	df	8
ART	(Intercept)	2.85E-02	4.88E-03	5.847	0.000384
	1HW	1.71E-02	9.21E-03	1.861	0.099753
	3HW	9.46E-03	8.95E-03	1.057	0.321415
		Null deviance:	1.32808	df	10
		Residual deviance:	0.87612	df	8

Table S2. Results of the GLM fitted to the ecosystem attributes. Significant increases of the treatment (1HW or 3HW) in relation to the 0HW are represented as green cells and marginally significant changes are highlighted in yellow cells.

Response variables	*Predictors*	*Estimates*	*Std. Error*	*t-value*	*Pr(>|t|)*
**GPP**	(Intercept)	3.73E-04	8.50E-05	4.392	0.00231
	1HW	-8.12E-05	1.08E-04	-0.753	0.47317
	3HW	9.70E-05	1.50E-04	0.646	0.53614
		Null deviance:	2.1072	df	10
		Residual deviance:	1.7184	df	8
**Respiration**	(Intercept)	9.68E-04	1.75E-04	5.546	0.000543
	1HW	-4.30E-04	2.00E-04	-2.152	0.063552
	3HW	-1.76E-04	2.40E-04	-0.734	0.484069
		Null deviance:	1.8474	df	10
		Residual deviance:	1.1289	df	8
**Export**	(Intercept)	-5.9262	0.2684	-22.077	1.87E-08
	1HW	-0.1378	0.3796	-0.363	0.726
	3HW	-0.5205	0.41	-1.269	0.24
		Null deviance:	2.8239	df	10
		Residual deviance:	2.3684	df	8
**Macrophytes biomass**	(Intercept)	2.89E-05	3.20E-06	9.021	1.82E-05
	1HW	-7.89E-07	4.47E-06	-0.176	0.864
	3HW	6.98E-06	5.60E-06	1.246	0.248
		Null deviance:	0.53655	df	10
		Residual deviance:	0.42356	df	8
**Consumers biomass**	(Intercept)	3.68E-04	3.30E-05	11.149	3.75E-06
	1HW	-6.22E-05	4.29E-05	-1.45	0.1851
	3HW	-9.66E-05	4.33E-05	-2.232	0.0561
		Null deviance:	0.39685	df	10
		Residual deviance:	0.23086	df	8
**Detritivory**	(Intercept)	6.48E-03	5.39E-04	12.026	2.11E-06
	1HW	-2.66E-03	6.25E-04	-4.261	0.00276
	3HW	-1.97E-03	6.91E-04	-2.859	0.0212
		Null deviance:	0.80057	df	10
		Residual deviance:	0.23698	df	8
**Herbivory**	(Intercept)	1.70E-02	2.21E-03	7.682	5.84E-05
	1HW	-2.48E-03	2.91E-03	-0.852	0.419
	3HW	-3.54E-03	2.99E-03	-1.184	0.27
		Null deviance:	0.63796	df	10
		Residual deviance:	0.53662	df	8

**D:H**	(Intercept)	3.59E-01	3.50E-02	10.255	7.03E-06
	1HW	-9.58E-02	4.33E-02	-2.21	0.0581
	3HW	-2.83E-02	5.10E-02	-0.553	0.5951
	Null deviance:		0.50363	df	10
	Residual deviance:		0.29552	df	8
**POC**	(Intercept)	2.89E-03	2.10E-04	13.731	7.63E-07
	1HW	3.80E-04	3.18E-04	1.195	0.266
	3HW	-5.76E-05	3.18E-04	-0.181	0.861
	Null deviance:		0.20495	df	10
	Residual deviance:		0.16038	df	8
**POC sink**	(Intercept)	1.40E-02	1.44E-03	9.721	1.05E-05
	1HW	-4.90E-03	1.72E-03	-2.844	0.0217
	3HW	-3.07E-03	1.94E-03	-1.579	0.1529
	Null deviance:		0.71886	df	10
	Residual deviance:		0.35278	df	8
